# Loss of hematopoietic progenitors heterogeneity is an adverse prognostic factor in lower-risk myelodysplastic neoplasms

**DOI:** 10.1038/s41375-024-02234-6

**Published:** 2024-04-04

**Authors:** Charles Dussiau, Thibault Comont, Camille Knosp, Inès Vergnolle, Clotilde Bravetti, Alban Canali, Amandine Houvert, Laetitia Largeaud, Christian Daveaux, Laila Zaroili, Chloé Friedrich, Ismaël Boussaid, Loria Zalmai, Carole Almire, Odile Rauzy, Lise Willems, Rudy Birsen, Didier Bouscary, Michaela Fontenay, Olivier Kosmider, Nicolas Chapuis, François Vergez

**Affiliations:** 1 Assistance Publique-Hôpitaux de Paris. Centre-Université Paris Cité, Service d’hématologie biologique, Hôpital Cochin, Paris, France; 2 Université Paris Cité, Institut Cochin, CNRSUMR8104, INSERM U1016, Paris, France; 3 Centre Hospitalier Universitaire de Toulouse, Institut Universitaire du Cancer de Toulouse Oncopole, Service de Médecine Interne, Toulouse, France; 4 Université Toulouse III Paul Sabatier, Toulouse, France; 5 Cancer Research Center of Toulouse, UMR1037-INSERM, ERL5294 CNRS, Toulouse, France; 6 Centre Hospitalier Universitaire de Toulouse, Institut Universitaire du Cancer de Toulouse Oncopole, Laboratoire d’Hématologie, Toulouse, France; 7 Assistance Publique-Hôpitaux de Paris. Centre-Université Paris Cité, Service d’hématologie clinique, Hôpital Cochin, Paris, France

**Keywords:** Myelodysplastic syndrome, Myelodysplastic syndrome, Translational research, Disease-free survival

## Abstract

Myelodysplastic neoplasms (MDS) are characterized by clonal evolution starting from the compartment of hematopoietic stem and progenitors cells (HSPCs), leading in some cases to leukemic transformation. We hypothesized that deciphering the diversity of the HSPCs compartment may allow for the early detection of an emergent sub-clone that drives disease progression. Deep analysis of HSPCs repartition by multiparametric flow cytometry revealed a strong disorder of the hematopoietic branching system in most patients at diagnosis with different phenotypic signatures closely related to specific MDS features. In two independent cohorts of 131 and 584 MDS, the HSPCs heterogeneity quantified through entropy calculation was decreased in 47% and 46% of cases, reflecting a more advanced state of the disease with deeper cytopenias, higher IPSS-R risk and accumulation of somatic mutations. We demonstrated that patients with lower-risk MDS and low CD34 + CD38+HSPCs entropy had an adverse outcome and that this parameter is as an independent predictive biomarker for progression free survival, leukemia free survival and overall survival. Analysis of HSPCs repartition at diagnosis represents therefore a very powerful tool to identify lower-risk MDS patients with a worse outcome and valuable for clinical decision-making, which could be fully integrated in the MDS diagnostic workflow.

## Introduction

Myelodysplastic neoplasms (MDS) represent an heterogeneous group of clonal disorders affecting hematopoietic stem and progenitor cells (HSPCs) [1]. Due to their inherent risk of progression to acute myeloid leukemia (AML), MDS constitutes a preleukemic condition that must be thoroughly investigated for each patient at diagnosis. Indeed, MDS display marked heterogeneity regarding the prognosis and the risk of disease progression which is assessed by the revised International Prognostic Scoring System (IPSS-R) [2]. The classifications of myelodysplastic neoplasms were recently revised and above all the IPSS-Molecular, a new clinical-molecular prognostic model has been proposed [3–5]. Indeed, the acquisition and expansion of additional driver mutations arising in HSPCs usually precedes further disease progression and can be therefore helpful to detect patients that may benefit from more aggressive treatment strategies.

Bone marrow (BM) of MDS patients are characterized by the progressive accumulation of HSPCs harboring molecular aberrations. However, HSPCs compartment includes a broad spectrum of cells at different stages of maturation in distinct lineages. This heterogeneity has been reported to be modified in case of MDS and aberrant phenotype of hematopoietic stem cells is associated with a higher risk of progression [6–12]. Interestingly, expansion of specific patterns of HSPCs is highly dependent of different signaling pathways which could be therapeutically targeted [13]. Thus, deciphering the heterogeneity of HSPCs in MDS may allow for the early detection of an emergent subclone that drives disease progression prior to clinical deterioration.

In this context, we designed a new MFC strategy based on the current hematopoietic hierarchical scheme [14, 15], to explore the hematopoietic branching system in MDS and integrate this strategy to the diagnostic workflow. Using a deep unsupervised analysis of HSPCs repartition, we identified a decreased diversity of HSPCs in most of MDS samples tested. The Shannon entropy used to measure HSPCs heterogeneity in two independent MFC datasets revealed that CD34 + CD38+HSPCs entropy was significantly decreased in a subset of MDS patients including at early stage of the disease. Interestingly, lower-risk MDS (LR-MDS) patients (IPSS-R ≤ 3.5) with an abnormal low level of CD34 + CD38+HSPCs entropy had an adverse outcome. We thus identify HSPCs entropy as a surrogate marker of clonal evolution in MDS patients underlying the adverse prognosis of MDS harboring a decreased HPSCs heterogeneity at diagnosis.

## Methods

### Patients and specimens

A total of 984 BM samples were collected between 2017 and 2022 in two different institutions (APHP.Center - Cochin Hospital - University Paris Cité, and Toulouse University Hospital). Supplementary Table 1 summarizes the type of samples and their repartition into 3 different cohorts. Morphological and cytogenetic analysis were performed for all patients with MDS suspicion and were used to classify patients as MDS according the WHO criteria or non-MDS. MDS prognosis was assessed using the IPSS-R [2]. All investigations were approved by the Institutional Review Boards and the procedures were in accordance with the Helsinki Declaration. See supplementary methods for details on selection of non-MDS samples.

### MFC analysis

MFC analysis were performed on CD34+ mononuclear sorted cells (with magnetic columns from Miltenyi Biotec™ MicroBead MACS technology) from thawed BM mononuclear cells previously isolated on a Ficoll gradient and stored in liquid nitrogen (cohort #1) and on fresh BM specimens (cohort #2 and cohort #3) (Supplementary Table 1). The different combination of antibodies used are described in Supplementary Table 2. All MFC analysis were performed blinded of morphological or cytogenetics results, and reciprocally. See supplementary methods for additional details.

### Genomic testing

Genomic DNA extracted from BM aspiration was studied by high throughput sequencing (HTS) of 45 genes recurrently mutated in myeloid malignancies as previously described [16]. See supplementary methods for additional details.

### Estimation of Shannon indexes

The Shannon entropy is a robust proxy that allows estimation of the diversity of a system. Based on the relative abundance of each HSPCs subpopulations identified by MFC, the Shannon entropy was computed among both CD34 + CD38+ and CD34 + CD38- compartment of HSPCs. Entropy is calculated as is: if H(X) be the HSPCs entropy, with pi the percentage of cells belonging to a subpopulation and k the number of subpopulations in the CD34 + CD38+ or CD34 + CD38- compartment:

$$H\left(X\right)=-\mathop{\sum }\limits_{i=1}^{k}{pi}\times \log 2({pi})$$

Mean normal rates of entropy H(N) and its standard deviation SD H(N) were calculated in non-MDS samples without cytopenia and were then used as a reference to analyze HSPCs entropy levels in patients with cytopenia related to MDS or not through z-score calculation (z = (H(X) – H(N)) / SD H(N)).

### Statistical methods

Heat map were obtained using the Morpheus analysis software (https://software.broadinstitute.org/morpheus) and hierarchical clustering were performed using the One minus pearson correlation metric with an average linkage method. T-SNE-FlowSOM workflow was performed using Cytobank. Progression-free (PFS) were defined according to standard IWG 2006 criteria (increase in percentage of bone marrow blasts ≥50% and progression to a more advanced MDS subtype than pretreatment) [17]. The follow-up of patients were censored at the time of last contact for overall survival (OS) and at the time of last contact or death without progression for PFS. Risk groups for prognosis were evaluated for OS and PFS by univariate analysis (log-rank test) and by a multivariate model of Cox regression or of Fine and Gray. All calculations were performed using STATA version 13 software (STATA Corp., College Station, TX), all graphs were drawn using Graph Pad Prism software (San Diego, CA) or R 3.6.1 (cran.rproject.org). Statistical test results are graphically expressed: *p < 0.05, **p < 0.01, ***p < 0.001, ****p < 0.0001.

## Results

### Heterogeneity of HSPCs is reduced in MDS

**Fig. 1:**
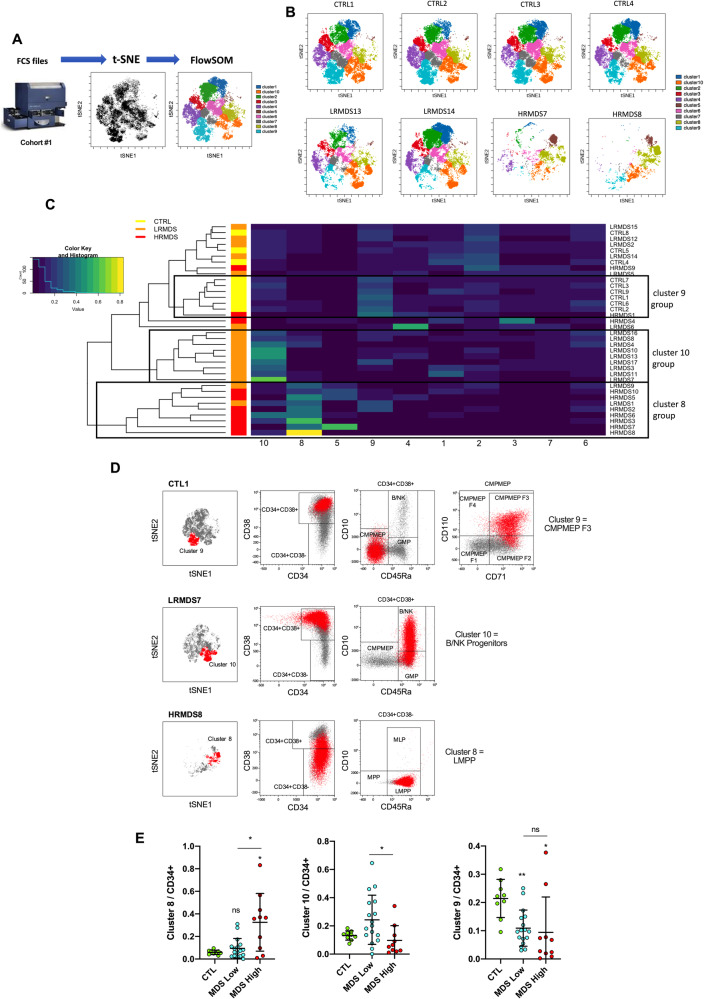
Deep immunophenotyping of HSPCs revealed a decreased heterogeneity of HSPCs in MDS samples. BM samples are from cohort #1. **A** Scheme of the pipeline used for automated clustering of CD34+ cell sorted subpopulations with the Cytobank web-based software. **B** CD34+ clusters repartition on a t-SNE plot from 4 representative control samples (CTL) and 4 MDS samples (low risk (LRMDS), *n* = 2; high risk (HRMDS), *n* = 2). **C** Heat map representation of clusters abundance among CD34+lin- sorted cells. Each column represents the relative frequency of the 10 clusters identified by the t-SNE-FlowSOM workflow for each sample (rows). The dendrogram demonstrates how the different MDS and control samples grouped together. Note that samples LRMDS1 and HRMDS2 come from serial BM aspiration of the same patient (each several months apart) and demonstrate consistent properties. **D** Phenotypic cells characterization of clusters 8, 9 and 10 in samples CTL1, LRMDS7 and HRMDS8, respectively. **E** Abundance of cluster 8, 9 and 10 in control (*n* = 9) and MDS samples according their low (*n* = 17) or high (*n* = 10) risk status. Lines represent means ± s.d. Statistical significance was determined using unpaired two-tailed Mann–Whitney tests.

We first investigated in details the distribution of CD34+lin-HSPCs in a cohort of MDS and non-MDS samples (cohort #1 in Supplementary Table 1) using a panel of 19 antibodies (combination #1 in Supplementary Table 2) allowing the identification of CD34+HSPCs sub-populations among both CD38- and CD38+ fractions of cells. The gating strategy and the complete phenotype of sub-populations identified is provided in Supplementary Fig. 1 and Supplementary Table 3 respectively. As expected, CD71 and CD110 expression led to the identification of different cell fractions (F1, F2 and F3) among multipotent progenitors (MPPs) and common myeloid/megakaryocyte-erythroid progenitors (CMPMEPs), which were previously reported to have distinct differentiation potential [14]. CD34+lin- cells from 27 MDS BM samples (Supplementary Table 4) and from 9 non-MDS samples without cytopenia matched for age were clustered using the pipeline summarized in Fig. 1A. The repartition on the t-SNE plot of the 10 clusters identified was highly similar between the control samples, whereas 6 among 17 patients with low-risk MDS and almost all high-risk MDS samples demonstrated distinct t-SNE patterns characterized by an accumulation of cells in a reduced number of clusters (Fig. 1B and Supplementary Fig. 2).

An automated hierarchical clustering of all samples according abundance of each clusters identified 3 groups of samples relatively homogeneous in terms of MDS or non-MDS conditions mainly according abundance of clusters 8, 9 and 10 (Fig. 1C). Interestingly, samples LRMDS1 and HRMDS2 which came from serial BM aspirations of a unique patient (12 months apart) demonstrated similar enrichment in clusters 8 and 9 at both low and high-risk stage, suggesting that abnormal HSPCs repartition pre-existed to disease progression. We identified the HSPCs sub-populations corresponding to cluster 8 as LMPPs, cluster 9 as CMPMEP F3 and cluster 10 as B/NK progenitors (Fig. 1D). The abundance of CMPMEP F3 and LMPPs were significantly modified in low- and/or high-risk MDS samples compared to control samples (Fig. 1E). B/NK progenitors and LMPP were respectively increased and decreased in low-risk compared to high-risk MDS (Fig. 1E).

Altogether, this indicates that a loss of HSPCs heterogeneity can be detected in most of MDS samples, including from early stage of these diseases.

### Different profiles of HSPCs repartition emerge in MDS

**Fig. 2:**
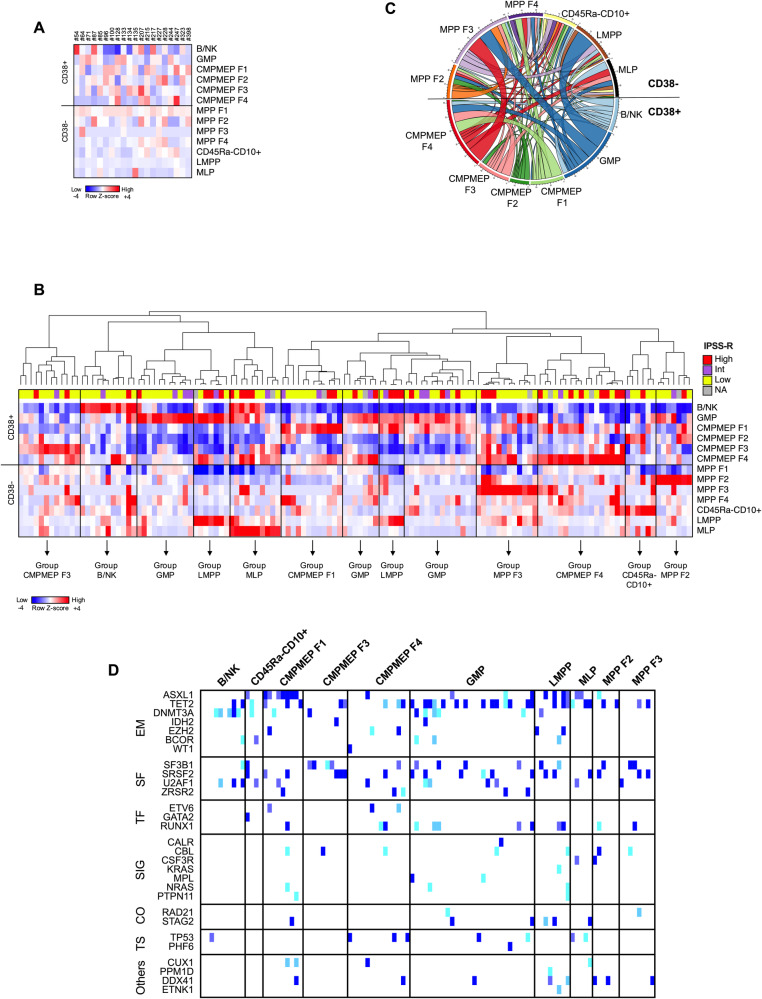
Identification of the different HSPCs repartition patterns in MDS. BM samples are from cohort #2. **A** Heatmap visualization of z-scores computed for all HSPC sub-populations (rows) from BM samples collected in patients with cytopenia not related to MDS (*n* = 20) compared to non-MDS samples without cytopenia. Each column in the heatmap is an individual sample. **B** Hierarchical clustering heat map visualization of z-scores computed for all HSPC sub-populations (rows) from BM samples collected in patients with MDS (*n* = 131) with their low (very low/low), intermediate or high (high/very High) IPSS-R score compared to non-MDS samples without cytopenia. Each column in the heatmap is an individual sample. The dendrogram demonstrates how the different MDS samples grouped together. NA, IPSS-R not available due to karyotype failure. **C** The circos diagram visualizes the association of the different phenotypic pattern of CD38+ and CD38-HSPCs in MDS samples in which abnormal accumulation of a least two HSPCs sub-populations was detected (*n* = 70). **D** Mutational landscape of the 102 MDS samples according the phenotypic HSPCs pattern. Mutational VAF is coded; ice, 2–10%; sky, 10–20%; orchid, 20–30%; blueberry, >30%. If multiple mutations are present in a case, the highest VAF is shown. EM epigenetic modifiers, SF splicing factors, SIG signaling, TF transcription factors, TS tumor suppressors.

We then applied a similar strategy of HSPCs identification with a 9 markers panel (combination #2 in Supplementary Table 2) to a large prospective cohort (cohort #2) of 184 fresh BM samples. The gating strategy and characteristics of MDS patients are described in Supplementary Fig. 3A, B and Table 1 respectively. We observed different types of abnormal HSPCs repartition in MDS samples such as accumulation of MLPs, LMPPs, MPP F2 (CD71 + CD110-), MPP F3 (CD71 + CD110 + ), GMPs, CMPMEP F2 (CD71 + CD110-), CMPMEP F3 (CD71 + CD110 + ) or decrease of GMPs and CMPMEP F3 (Supplementary Fig. 4A). Some sub-populations of HSPCs not observed in control samples such as CD34 + CD38-CD45Ra-CD10+ cells or MPP or CMPMEP CD110 + CD71- (called thereafter MPP F4 and CMPMEP F4) were also detected in MDS (Supplementary Fig. 4B), suggesting that the hematopoietic hierarchy could be impaired. We quantified the proportion of each CD34 + CD38+ and CD34 + CD38-HSPCs sub-populations detected in non-MDS samples without cytopenia (Supplementary Table 5) and this repartition was further used as a reference to analyze HSPCs distribution in BM samples from patients with cytopenia related or not to MDS through z-score calculation. Only few differences were observed in the HSPCs repartition in BM samples from patients with cytopenia unrelated to MDS (Fig. 2A), whereas HSPCs repartition was significantly more frequently abnormal in MDS patients (Fig. 2B) (7/260 (2.7%) vs 412/1703 (24.2%) abnormal z-score values <-2 or>2 respectively, *p* < 0.0001). GMPs, CMPMEP F4 and MPP F3 were the most frequently increased HSPCs sub-populations in MDS samples (Supplementary Fig. 4C). Hierarchical clustering identified distinct group of MDS samples with profiles of HSPCs repartition with increase of specific HSPCs sub-populations frequently correlated with IPSS-R stratification (Fig. 2B). Profiles with accumulation of LMPPs or MLPs were enriched in patients with high-risk MDS, whereas the accumulation of B/NK, GMPs or CMPMEP F3 was more frequently related to low-risk MDS (Fig. 2B). Accordingly, profiles with accumulation of LMPPs or B/NK progenitors were characterized by the highest and the lowest median blast cells count respectively (Supplementary Fig. 4E). Analysis of the dependency of each phenotypic pattern revealed association of not only LMPPs with GMPs, but also GMPs with MPP F3 and CMPMEP F4 with either MPP F3, CMPMEP F1 or GMP (Fig. 2C).

**Table 1:** Presenting features of patients with MDS from cohort #2 and cohort #3.

Variables	Cohort #2 (Institution #1)				Cohort #3 (Institution #2)			
	All patients (*n* = 131)	CD38 + HSPCs Entropy			All patients (*n* = 584)	CD38 + HSPCs Entropy		
		Normal (*n* = 69, 53%)	Low (*n* = 62, 47%)	*p* value		Normal (*n* = 318, 54%)	Low (*n* = 266, 46%)	*p* value
Age, y, median (range)	76 (29–94)	75 (42–89)	77 (29–94)	0.6366	74 (19–96)	75 (23–95)	72 (19–96)	**0.022**
Males, n (%)	78 (60)	41 (59)	37 (60)	1	237 (40.6)	128 (40.3)	109 (41)	0.87
Hb, g/dL, median (range)	10.7 (6.7–15.4)	10.7 (7.9–15.4)	10.75 (6.7–15.4)	0.9669	9.3 (3.3–15.2)	9.4 (3.3–15.2)	9.1 (5.3–15.2)	0.19
Hb < 10 g/dL, n (%)	43 (33)	24 (32)	19 (31)	0.7101	363 (62)	192 (60)	171 (64)	0.35
Hb < 8 g/dL, n (%)	12 (9)	2 (3)	10 (16)	**0.013**	124 (21)	61 (19)	63 (24)	0.19
ANC, x 10^9^/L, median (range)	1.98 (0.03–12.24)	2.74 (0.67–8.64)	1.44 (0.03–12.24)	**<0.0001**	1.65 (0.06–9.51)	1.76 (0.06–9.51)	1.43 (0.10–9.32)	0.073
ANC < 0.8 × 10^9^/L, n (%)	16 (12)	2 (3)	14 (27)	**0.0008**	133 (23)	66 (21)	67 (25)	0.23
Plt, x10^9^/L, median (range)	132 (21–584)	156 (28–453)	116 (21–584)	**0.0028**	90 (5–1028)	101 (5–892)	83 (5–1028)	**0.0015**
Plt < 100 × 10^9^/L, n (%)	41 (31)	15 (22)	26 (42)	**0.0228**	324 (55)	158 (50)	166 (62)	**0.0026**
Plt < 50 × 10^9^/L, n (%)	7 (5)	2 (3)	5 (8)	0.2572	166 (28)	75 (24)	91 (34)	**0.0056**
Pancytopenia, n (%)	33 (25)	10 (15)	23 (37)	**0.0044**	115 (20)	53 (17)	62 (23)	**0.0476**
BM blast %, median (range)	3 (0–19)	2 (0–11)	5.5 (1–19)	**<0.0001**	10 (0–19)	4 (0–19)	10 (0–19)	**<0.0001**
Abnormal Karyotype, n (%)	47 (38)	25 (37)	22 (39)	0.8538	325 (56)	167 (53)	158 (59)	0.11
Very good	9 (7)	7 (10)	2 (4)	**0.0109**	24 (4)	14 (4)	10 (4)	0.55
Good	83 (68)	46 (69)	37 (66)		306 (52)	176 (55)	130 (49)	
Intermediate	15 (12)	6 (9)	9 (16)		107 (18)	52 (16)	55 (21)	
Poor	6 (5)	6 (9)	0 (0)		59 (10)	31 (10)	28 (11)	
Very Poor	10 (10)	2 (3)	8 (14)		88 (15)	45 (14)	43 (16)	
NA	8	2	6		0	0	0	
WHO 2022, n (%)								
MDS-LB	63 (48)	43 (62)	20 (32)	**<0.0001**	126 (22)	98 (31)	28 (11)	**<0.0001**
MDS-LB-RS	8 (6)	7 (10)	1 (2)		38 (7)	27 (8)	11 (4)	
MDS-5q	3 (2)	2 (3)	1 (2)		22 (4)	14 (4)	8 (3)	
MDS-IB1	24 (18)	11 (16)	13 (21)		108 (18)	49 (15)	59 (22)	
MDS-IB2	16 (12)	0	16 (26)		218 (37)	87 (27)	131 (49)	
MD-CMML1	14 (11)	5 (7)	9 (15)		48 (8)	32 (10)	16 (6)	
MD-CMML2	3 (2)	1 (2)	2 (3)		24 (4)	11 (3)	13 (5)	
IPSS-R, n (%^b^)								
NA	8	2	6		0	0	0	
Very Low	27 (22)	23 (34)	4 (7)	**<0.0001**	53 (9)	42 (13)	11 (4)	**<0.0001**
Low	46 (37)	30 (45)	16 (29)		125 (21)	79 (25)	46 (17)	
Intermediate	17 (14)	3 (5)	14 (25)		119 (20)	67 (21)	52 (20)	
High	24 (20)	10 (15)	14 (25)		151 (26)	67 (21)	84 (32)	
Very High	9 (7)	1 (1)	8 (14)		136 (23)	63 (20)	73 (27)	
Mutated cases, n (%)	85 (83)^a^	40 (73)	45 (96)	**0.0026**	NA			
Number of mutations, median (range)	2 (0–10)	2 (0–7)	3 (0-10)	**<0.0001**	NA			
IPSS-M, n (%^c^)					NA			
Very Low	12 (12)	10 (18)	2 (5)	**0.0007**				
Low	45 (46)	32 (58)	13 (30)					
Moderate Low	4 (4)	2 (4)	2 (5)					
Moderate High	15 (15)	6 (11)	9 (20)					
High	13 (13)	4 (7)	9 (20)					
Very High	10 (10)	1 (2)	9 (20)					

Comparisons were performed using a Mann–Whitney test for continuous variables and Fisher’s exact tests for categorical variables. Significant *p* values are highlighted in bold.

*ANC* absolute neutrophils count, *BM* bone marrow, *Hb* hemoglobin, *MD-CMML1/2* myelodysplastic-chronic myelomonocytic leukemia 1/2 (WBC < 13 × 109/L), *MDS-5q* MDS with low blasts and with isolated del(5q), *MDS-IB1/2* MDS with increased blasts 1/2, *MDS-LB* MDS with low blasts, *MDS-LB-RS* MDS with low blasts and ring sideroblasts, *NA* not available, *Plt* platelet.

^a^Cases with HTS data, *n* = 102.

^b^Among cases with IPSS-R available.

^c^Cases with HTS & cytogenetic data &, *n* = 99.

We also analyzed the genetic landscape of 102/131 MDS patients of the cohort #2 according to their phenotypic pattern of HSPCs repartition. Overall, 85/102 (83.3%) patients carried at least one somatic mutation (Table 1). Patients with an increase of B/NK progenitors or CMPMEP F3 or MPP F3 harbored the lowest number of mutations, whereas those with an increase of MPP F2, MLPs or LMPPs were associated with a higher number of mutations (Supplementary Fig. 4E). Interestingly, the profile with increased of B/NK progenitors was associated with DNMT3A mutations (5/10 patients, *p* = 0.0058), whereas SF3B1 mutations were more frequent in patients with increased of CMPMEP F3 (4/10 patients; *p* = 0.0482) (Fig. 2D). The profile with accumulation of CMPMEP F1 was also frequently associated with ASXL1 mutation (7/9 patients; *p* < 0.0001) and TET2 mutations were more frequently detected in patients with increase of GMPs (15/28 patients; *p* = 0.0217) (Fig. 2D).

Altogether, these results suggested the amplification of distinct HSPCs sub-populations in MDS and revealed phenotypic signatures associated with IPSS-R stratification and distinct genomic patterns of MDS.

### MDS with decreased CD38 + HSPCs heterogeneity have a reduced entropy

**Fig. 3:**
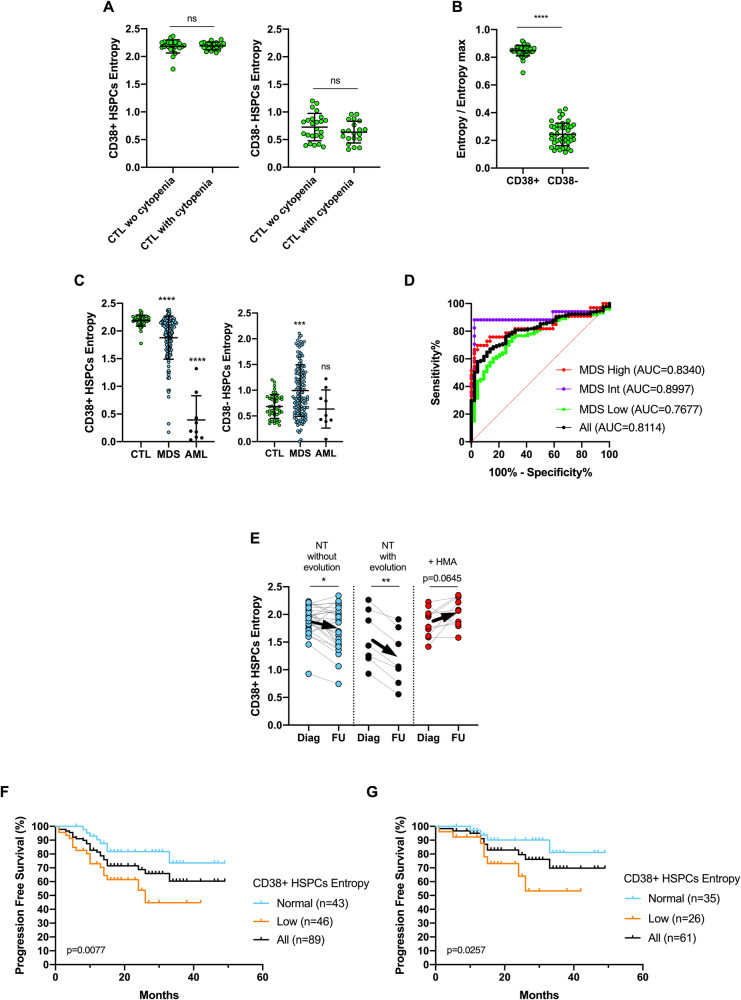
Reduced HSPCs heterogeneity in MDS can be assessed by a decreased entropy of HSPCs. BM samples are from cohort #2. **A** Entropy levels in both CD34 + /CD38- and CD34 + /CD38+ fractions of HSPCs in non MDS samples (CTL) without cytopenia (*n* = 24, left panel) or with cytopenia (*n* = 20, right panel). Lines represent means ± s.d. Statistical significance was determined using unpaired two-tailed Mann–Whitney tests. **B** Comparison of CD38+ and CD38-HSPCs entropy value in non MDS-samples with or without cytopenia. Given the different number of HSPCs sub-populations identified in the CD34 + CD38+ (*n* = 6) and the CD34 + CD38- (*n* = 7) fraction of HSPCs, entropy values were normalized to the maximum possible entropy rate (2.58 for CD38+HSPCs and 2.81 for CD38-HSPCs) to allow comparison of entropy rates in these two compartment of cells. Lines represent means ± s.d. Statistical significance was determined using Wilcoxon matched-pairs signed rank tests. **C** Comparison of CD38+ (left panel) and CD38- (right panel) HSPCs entropy levels in non-MDS samples (with or without cytopenia) (*n* = 44), in MDS samples (*n* = 131) and in AML samples (*n* = 9). Lines represent means ± s.d. Statistical significance was determined using unpaired two-tailed Mann–Whitney tests. **D** ROC curve for the CD38+HSPCs entropy in the whole cohort of patients with MDS (black curve, *n* = 131), or MDS low risk (very low/low IPSS-R; orange curve; *n* = 73) or MDS intermediate risk (blue curve; *n* = 17) or MDS high risk (high/very high IPSS-R; red curve; *n* = 33) compared to non-MDS samples with or without cytopenia (*n* = 44). The Youden index was defined for all points of the ROC curve and the maximum value of the index (152.54) was used as a criterion for selecting the optimum cutoff point of the CD38+HSPCs entropy (2.1) to identify patients with MDS. **E** Dynamic of CD38+HSPCs entropy during the follow-up of untreated low-risk MDS patients with stable disease (based on a stable BM blast cell count) (*n* = 24; median follow-up of 11.5 months ; range: 3–40 months); or with evolution attested by increased BM blast cell count (*n* = 7; median time between diagnosis of MDS and evolution of the disease: 12 months ; range : 9–26 months) and of MDS patients treated by HMA leading to stabilization of the disease based on the BM blast cell count (*n* = 10; median number of cures: 6 ; range : 2–12). Lines represent means ± s.d. Statistical significance was determined using Wilcoxon matched-pairs signed rank tests. Black arrows indicate the evolution of the mean value of CD38+HSPCs entropy between diagnosis (Diag) and follow-up (FU). Progression Free Survival curves (**F**) of MDS patients whatever the IPSS-R classification (*n* = 84) or (**G**) of patients with LR-MDS (defined as IPSS-R ≤ 3.5) (*n* = 61), according to the low or normal level of CD38+HSPCs entropy (z-score ≤-2 or >-2 compared to non-MDS samples without cytopenia).

To quantify the heterogeneity of HSPCs in the BM, we computed the Shannon entropy based on quantification of the proportion of each HSPCs subpopulations identified by MFC in samples from the cohort #2. Entropy of CD34 + CD38+ and CD34 + CD38-HSPCs was similar in non-MDS samples with or without cytopenia (Fig. 3A). Comparison of entropy values normalized to the maximum possible entropy level of these two fractions of cells showed a significantly lower CD38-HSPCs entropy, which reflects the lower phenotypic diversity of immature multipotent CD38- progenitors compared to more differentiated CD38+HSPCs (Fig. 3B). The entropy of CD38+HSPCs was significantly decreased in MDS samples and also drastically reduced in AML samples (Fig. 3C). Conversely, the CD38-HSPCs entropy was significantly increased in MDS samples (Fig. 3C), attesting the emergence and/or accumulation of one or more CD38-HSPCs sub-populations, creating a heterogeneity not observed in non-MDS samples. In AML samples, the level of CD38-HSPCs entropy remained low, mainly due to the full replacement of normal CD34 + CD38- HSPCs by abnormal CD34 + CD38- leukemic stem cells.

Normal rates of CD38+ and CD38-HSPCs entropy in non-MDS samples without cytopenia were then used as a reference to analyze HSPCs entropy levels in patients with cytopenia related to MDS or not. Whereas CD38+HSPCs entropy was normal in all non-MDS samples with cytopenia, a decrease of CD38+HSPCs entropy (z-score ≤-2) was found in 47.3% of MDS cases (Supplementary Fig. 5A). Increase of CD38-HSPCs entropy (z-score ≥2) was also detected in MDS samples but to a significantly lower frequency (32.8% of cases; *p* = 0.0230) (Supplementary Fig. 5A). With a ROC analysis, we identified a CD38 + HSPC entropy value < 2.1 allowing the detection of MDS with 86.3% of specificity and 67.2% of sensitivity with higher AUC for intermediate and high-risk MDS (Fig. 3D). However, the increase of CD38-HSPCs entropy was less efficient to diagnose MDS (Supplementary Fig. 5B).

Altogether, these data indicated therefore that HSPCs entropy and more specifically the CD38+HSPCs entropy is a relevant parameter for the diagnosis of MDS.

### Reduced CD38 + HSPCs entropy identified patients with specific MDS features

We then investigated whether CD38+HSPCs entropy correlated with clinical and biological features of MDS cases (Table 1). Age was similar in the two groups of MDS patients. A low level of CD38+HSPCs entropy was associated with lower neutrophils and platelets counts, deep anemia and pancytopenia (Table 1). The level of CD38+HSPCs entropy was inversely correlated with CD34+ cells quantified by MFC and with the BM blast count assessed by morphology (Supplementary Fig. 5C). Accordingly, low CD38+HSPCs entropy was associated with the MDS-IB entity of the WHO classification, a poor risk cytogenetic profile and a very high/high/intermediate risk IPSS-R (Table 1). We also analyzed the genetic landscape of MDS patients according to the level of the CD38+HSPCs entropy (Supplementary Fig. 6A). Abnormal CD38+HSPCs entropy was associated with increased proportion of mutated cases and a higher number of mutations (Table 1). Low level of CD38+HSPCs entropy was significantly more frequently associated with somatic mutations in genes encoding for transcription factors (*p* = 0.0144, Supplementary Fig. 6B). Among these genes, RUNX1 was the most frequently mutated in patients with low CD38+HSPCs entropy (*p* = 0.0109). ASXL1 (*p* = 0.0412) and DDX41 (*p* = 0.0228) mutations were also more frequently detected in patients with abnormal CD38+HSPCs entropy (Supplementary Fig. 6C). Interestingly, we detected in MDS patients harboring a somatic mutation with high variant allele frequency (VAF), a lower CD38+HSPCs entropy (Supplementary Fig. 6D), suggesting that evaluation of HSPCs entropy could reflect the clonal dominance observed in MDS. We also analyzed the association between the level of CD38+HSPCs entropy and patient restratification according to the IPSS-M computed based on these molecular data. The restratification of IPSS-R to IPSS-M was as expected (Supplementary Fig. 7A, B). Low CD38+HSPCs entropy was significantly associated with a moderate high/high/very high IPSS-M (Table 1). Interestingly when focusing on patients which were restratified upstaged, 13/31 (42%) of cases were characterized by low level of CD38+HSPCs entropy with higher proportion in the group of low, intermediate or high IPSS-R (Supplementary Fig. 7C). Furthermore, among patients with LR-MDS based on the IPSS-R ( ≤ 3.5), 5/7 (71%) of cases restratified in the group of HR-MDS based on the IPSS-M (>0) harbored a low level of CD38+HSPCs entropy (Supplementary Fig. 7D). These results suggest therefore that this parameter could allow the identification of some patients who will be restratified upstaged with the IPSS-M classification, especially those who will switch from a LR-MDS to a HR-MDS.

We also followed the level of CD38+HSPCs entropy during the course of the disease. In untreated patients with a stable low-risk MDS, the CD38+HSPCs entropy significantly decreased during follow-up and this was more pronounced in patients with disease progression (Fig. 3E). However, this decrease was no longer observed in patients with hypomethylating agents-mediated stabilization of the disease (Fig. 3E). Finally, we observed that MDS patients with an abnormal low level of CD38+HSPCs entropy computed at diagnosis had a significantly higher risk of progression and this was also true for the sub-group of patients with LR-MDS (Fig. 3F, G). Altogether, these results indicated that the detection of a loss of CD38+HSPCs heterogeneity through entropy calculation is an attractive biomarker for predicting the outcome of MDS patients, including from early stage of the disease and for monitoring their follow-up.

### Validation of CD38 + HSPCs entropy as a diagnostic tool for MDS detection

To validate the importance of CD38+HSPCs entropy in the diagnosis of MDS, we took advantage of an independent cohort of 764 patients (cohort #3 in Supplementary Table 1 and Table 1). The panel markers (combination #3, Supplementary Table 2) and the analysis strategy (Supplementary Fig. 8) we used for these samples led to the identification of 6 different CD38+HSPCs subpopulations (Supplementary Table 3). We confirmed in this cohort that the level of CD38+HSPCs entropy was similar in non-MDS samples with or without cytopenia, and significantly decreased in MDS samples (Supplementary Fig. 9A). ROC curve evaluation confirmed the capacity of CD38+HPSCs entropy to diagnose MDS including for low-risk MDS (Supplementary Fig. 9B). Samples from non-MDS patients without cytopenia were then used to define the normal level of CD38 + HSPC entropy and calculation of a z-score < -2 identified 198 (46%) MDS patients with an abnormal low level of CD38+HSPCs entropy (Supplementary Fig. 9C). Similarly, the decreased CD38+HSPCs entropy level was associated with specific features of MDS such as deeper thrombocytopenia and neutropenia (Table 1). CD38 + HSPC entropy of MDS patients was closely related to the level of BM blasts with a negative linear correlation (Supplementary Fig. 9D). Consistent with this, low level of CD38+HSPCs entropy was also associated with the “MDS with increased blasts” entity of the WHO classification and with the high/very high-risk IPSS-R (Table 1).

These results confirmed therefore in this independent cohort, the capacity of CD38+HSPCs entropy calculation to identify MDS patients with a more advanced stage of their disease.

### Low CD38 + HSPCs entropy is an independent negative prognostic marker for MDS patients

**Fig. 4:**
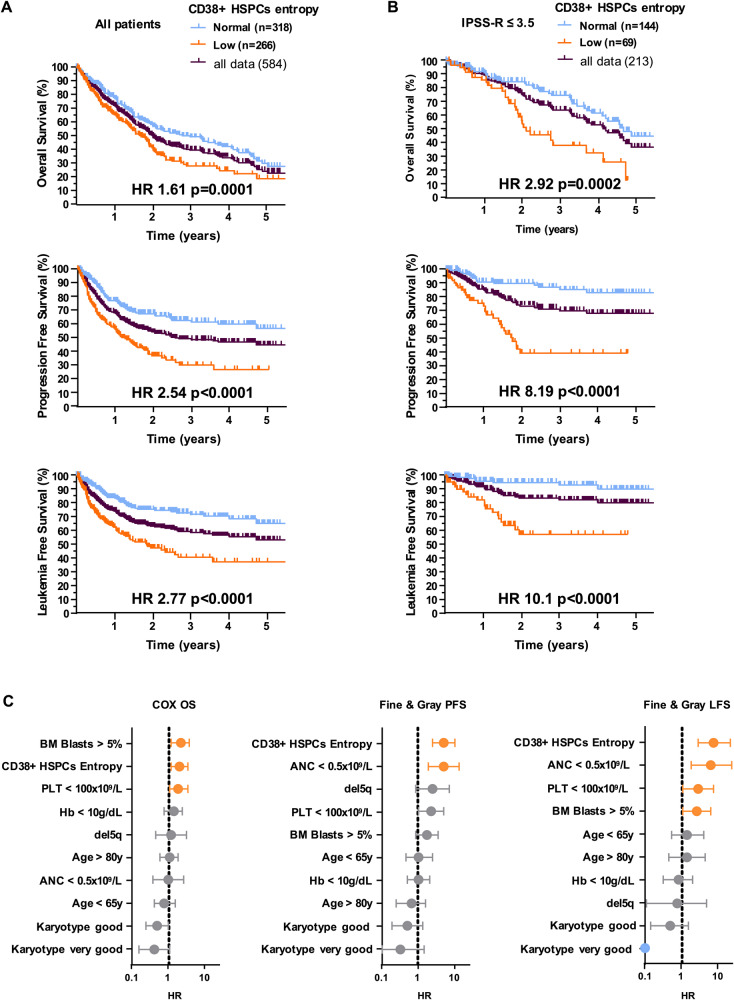
Low level of CD38 + HSPCs is an independent negative prognostic factor for LR-MDS. BM samples are from cohort #3. Kaplan-Meier estimates of survival outcomes according to CD38+ HSPCs entropy level of (**A**) all patients (*n* = 584) or (**B**) of patients with LR-MDS (IPSS-R ≤ 3.5) (*n* = 213). **C** Multivariate analysis exploring factors that affect PFS, LFS and OS of LR-MDS patients (*n* = 213). Good or very good cytogenetic risk categorization was determined according the Comprehensive Cytogenetic Scoring System (CCSS) for MDS.

Finally, we wondered whether the CD38 + HSPCS entropy could be an independent prognostic parameter in MDS. We thus analyzed the follow-up of 584 MDS patients (median follow-up of 28.4 months) classified according their low or normal level of CD38+HSPCs entropy computed at diagnosis. The impact of the IPPS-R classification on LFS and OS was as expected (Supplementary Fig. 9E). Interestingly, the median PFS of patients with low CD38+HSPCs entropy was significantly decreased (14.9 months vs not reached (NR); Hazard Ratio [HR]:2.40, *p* < 0.0001) (Fig. 4A). The median LFS of patients with low CD38+HSPCs entropy was also significantly decreased (22.1 months vs NR; HR:2.62, *p* < 0.0001) (Fig. 4A). One-year (y) and 3-y LFS were respectively of 64% and 40% in the low entropy patient’s group and 83% and 72% for patients with normal entropy (Fig. 4A). The median OS of patients with a low CD38+HSPCs entropy was also significantly shorter (19.8 vs 33.2 months; HR:1.58, *p* = 0.0001) (Fig. 4A). One-y and 3-y OS were respectively 69% and 28% in low entropy patients and 78% and 49% in normal entropy patients (Fig. 4A). Interestingly, among the group of patients with LR-MDS (defined with IPSS-R ≤ 3.5), CD38+HSPCs entropy was still significantly associated to a shorter PFS (HR: 5.35; 95% confidence interval [95%CI]: 2.73–10.47, *p* < 0.0001), LFS (HR: 6.89; 95%CI: 2.98–15.91, *p* < 0.0001) and OS (HR: 2.37; 95%CI: 1.36–4.10, p = 0.0019) (Fig. 4B). Notably, LFS at 1 and 3 years were 96% and 93% respectively for MDS with normal entropy, compared to 82% and 57% for patients with low CD38+HSPCs entropy. In contrast, no or low significant differences of OS (*p* = 0.76), PFS (HR: 1.46; 95%CI: 1.07–2.00, *p* = 0.019) and LFS (HR: 1.59; 95%CI: 1.13–2.23, *p* = 0.0077) were observed between patients with low or normal level of CD38+HSPCs entropy among the group of patients with higher-risk MDS (HR-MDS) (Supplementary Fig. 10). In light of these results, we then tested whether the CD38+HSPCs entropy was an independent predicting factor for adverse outcome of these patients with LR-MDS. With a multivariate Fine and Gray analysis, low CD38+HSPCs entropy was found to be strongly independently associated with a shorter PFS (HR = 5.21; 95%CI: 2.58–10.51, *p* < 0.0001) and a shorter LFS (HR = 7.77; 95%CI: 2.81–21.45, *p* < 0.0001) (Fig. 4C). In multivariate Cox analysis, low CD38+HSPCs entropy was also significantly associated with a worse OS (HR = 2.03; 95%CI: 1.17–3.51, *p* = 0.012) (Fig. 4C).

Altogether, these results confirmed that decreased CD38+HSPCs entropy is a strong predictor of adverse outcomes of LR-MDS independently of others parameters included in the IPSS-R risk stratification.

## Discussion

Diagnosis and prediction of MDS evolution remain challenging in a daily clinical practice. Our increasing understanding of the molecular mechanisms that drive the pathophysiology of MDS recently led to a new clinical-molecular prognostic model, which allows a more precise evaluation of outcomes [4]. However, it remains unclear at this time how to address the reality that genomic analysis by high throughput sequencing are complex and not yet worldwide used. Improving prognostic systems with new biomarkers based on MDS physiopathology which can be rapidly and easily collected could allow a more precise and fast risk stratification in most healthcare facilities. MFC, an indispensable tool for providing key information for the diagnosis and the monitoring of almost all hematopoietic neoplasms, can also provide key information not only for MDS diagnosis [18–22] but also for guiding the choice of the therapeutic strategy [13, 23, 24] and for predicting the risk of progression [12, 13, 19, 25]. Analysis of the HSPCs compartment which contains the cell of origin for MDS represents an attractive strategy to appreciate, at diagnosis, the state of advancement of the disease and to evaluate how close or far the leukemic transformation is. We thus designed a new MFC application to explore the hematopoietic branching system which can be fully integrated in the diagnostic workflow of MDS.

Here, we provide clear evidences that deep analysis of HSPCs repartition by MFC, reveals a reduced HSPCs heterogeneity in many MDS samples at diagnosis. Distinct abnormal profiles of HSPCs repartition in MDS samples were observed, some of them already reported such as the accumulation of GMPs, CMPs or LMPPs [9, 11, 13]. However, we also identified new profiles characterized by the accumulation of B/NK progenitors or MLPs reflecting a strong disorder of the hematopoietic branching system. Interestingly, these signatures seem to be closely related to specific IPSS-R risk but also to distinct molecular pattern of somatic mutations. Indeed, some phenotypic and genotypic associations we observed are in accordance with previous work such as the increased of CMPMEP F3 subpopulation with SF3B1 mutation given the enrichment of SF3B1mut cells in the megakaryocytic-erythroid lineage [26], or the increase of GMP in patients with TET2 mutations given its well-known effect toward the granulomonocytic differentiation of human hematopoietic progenitors [27]. This suggests therefore, that deciphering the HSPCs architecture could reflect distinct lineage trajectories that could be driven by specific mutations.

Computed Shannon entropy, a popular metric commonly employed in diverse scientific fields to assess the heterogeneity of a system [28, 29], allowed an accurate quantification of HPSCs repartition evidenced by MFC. Herein, we demonstrate in two cohorts of MDS patients that the CD38+HSPCs entropy is frequently decreased in MDS samples (47% and 46% of cases), reflecting the expansion of one or more CD38+HSPCs subpopulations and a more advanced state of the disease. However, low CD38+HSPCs entropy is still observed in patients with LR-MDS identified on either the IPSS-R or the IPSS-M classification, underlying the relevance of this parameter for the diagnosis of all MDS. The CD38-HSPCs compartment is also highly relevant since we observed in some MDS samples an abnormally elevated CD38-HSPCs entropy due to the emergence and/or accumulation of one or more CD38-HSPCs sub-populations. However, the very low level of CD38-HSPCs entropy computed in non-MDS samples is probably related to the panel of markers we used which did not allow the identification of either MPPs and HSCs. A more detailed analyze of CD38-HSPCs phenotype should probably increase the expected value of entropy in this compartment of cells and this should be investigated in the context of MDS.

Progression of MDS is currently mostly predicted by the genomic status detected at diagnosis. However, we demonstrate herein, that the detection of a reduced CD38+HSPCs heterogeneity attested by a low entropy at diagnosis is a robust biomarker to predict the worse outcome of LR-MDS patients. Multivariate analysis confirmed that the CD38+HPSCs entropy is an independent predictive biomarker for PFS, LFS and OS in this subgroup of MDS patients. Analysis of cellular HSPCs architecture represents therefore a very powerful tool to identify LR-MDS patients with a high risk of progression. This tool could be easily integrated in the workflow of MDS diagnosis for guiding patient care. Furthermore, we observed a decreased of CD38+HSPCs entropy during the follow-up of patients whose disease progressed but also in untreated patients without apparent evolution, indicating that CD38+HSPCs entropy is highly sensitive to detect MDS evolution. In contrast, HMA treatment leading to disease stabilization avoids this progressive trajectory of CD38+HSPCs entropy. Finally, at the time of refining myeloid diseases classification [3, 30], the frontier between MDS and AML trends to be less clear, considering that therapeutic strategies such as venetoclax-based therapy are now frequently used not only in AML but also in MDS/AML. Given the potential of such therapy for eradicating MDS/leukemic stems cells [16], monitoring the HSPCs compartment and detecting the persistence of MDS/leukemic stem cells like in AML [31] could be useful for attesting the efficiency of these treatments.

From a technical point of view, it is highly relevant to see that similar results were observed in two independent cohorts with MFC data obtained with different combinations of markers. Indeed, the median level of CD38+HSPCs entropy was similar in both cohorts due to the identification of the same type and number (n = 6) of sub-populations. Furthermore, this strategy is based on the identification and quantification of HSPCs sub-populations using a single-tube panel strategy and not on the detection of an abnormal profiles of maturation which are sometimes difficult to assess. In addition, this panel also allows the calculation of other well-known FCM based scores for MDS diagnostic such as the Ogata-Score or the Red-Score. This strongly supports therefore the feasibility of integration of the quantification of HSPCs repartition into the routine analysis of BM samples with MDS suspicion. Furthermore, investigation of both molecular patterns and phenotypic profiles have to be performed in parallel in larger cohorts of patients, in order to define more robust correlations between specific HSPCs signatures and patterns of mutations, especially those which are now included in the new IPSS-M risk stratification [4]. Multi-centric studies are therefore mandatory to confirm the correlation of CD38+HSPCs entropy with specific characteristics of MDS and their evolution and to determine how this parameter could be integrated among the already world-wide used IPSS-R stratification or the more recently developed IPSS-M.

Altogether, we demonstrate that the monitoring of HPSCs entropy should be considered as a highly sensitive biomarker for predicting outcomes of LR-MDS patients at diagnosis but also to detect progression during the course of the disease and propose it as a new tool for precision medicine.

### Supplementary information

Supplemental material

## Data Availability

The data that support the findings of this study are available from the corresponding author upon reasonable request.
