# Supplementary material for: Loss of hematopoietic progenitors heterogeneity is an adverse prognostic factor in lower-risk myelodysplastic neoplasms
Source: Leukemia. 2024 Apr 4;38(5):1131–42. doi: 10.1038/s41375-024-02234-6 (PMC11073965; doi:10.1038/s41375-024-02234-6)
Supplement: Supplementary file 1 — Supplemental material [file 41375_2024_2234_MOESM1_ESM.pdf]

Loss of hematopoietic progenitors heterogeneity is an adverse prognostic factor in lower-risk myelodysplastic syndromes

Dussiau C *et al*

Supplementary Material

.

SUPPLEMENTAL METHODS

Patients and specimens

BM control samples of cohort #1 (n=9) were from healthy elderly patients (median age of 68 years; min-max: 45-83) obtained from the bone of the femoral head obtained after informed consent, during a surgery for hip replacement. This non-interventional study was approved by the ethical committee of Cochin-Port Royal Hospital (CLEP Decision N°: AAA-2020-08039). In the cohort #2 and #3, non-MDS samples without cytopenia (cohort #2: n=24; median age of 67 years, min-max: 28-91 and cohort #3: n=54; median age of 61 years, min-max: 32-91) were from patients referred to our institutions for BM aspiration in a context of suspicion of different hematological neoplasms (mastocytosis, lymphoma or myeloma) without excess of abnormal cells. Non-MDS samples with cytopenia (cohort #2: n=20, median age of 70 years, min-max: 21-90 and cohort #3; n=126 median age of 70 years, min-max: 21-90) were from patients for who a diagnosis of MDS was excluded based on morphological analysis and/or cytogenetic data and/or to identification of non-MDS related cause.

MFC analysis

MFC analysis for samples of cohort #1 (Supplemental Table 1) using combination #1 of antibodies (Supplemental Table 2) were performed on BM mononuclear cells (BMMNCs) from sternal aspirations or from femoral heads previously isolated on a Ficoll gradient and stored in liquid nitrogen at a concentration of 20 to 30 million cells per mL. The femoral heads were cut in half and collected in a conservation medium (Hanks balanced salt solution with NaHCO3, Eurobio™), supplemented with heparin (7%) and then transported to the laboratory at room temperature. These were scraped with a spatula, grounded in a mortar, and washed with a PBS solution supplemented with DNAse (Sigma Aldrich) at 100ug/mL. BMMNCs were thawed in Iscove's modification of Dulbecco medium supplemented with DNAse (1ug/mL) to avoid the formation of aggregates and limit cell loss. BMMNCs were washed with PBS and sorted on magnetic columns (Miltenyi Biotec™ MicroBead MACS technology) to recover CD34 positive cells. CD34 positive cells were incubated for 20 min in Brilliant Stain Buffer, BD Horizon™ (100µL per tube) with the combination of antibodies shown in supplemental Table 2. Cells were washed with PBS (Dulbecco's PBS™ buffer) and a second labeling was performed with Zoombie acqua Amcyan (BioLegend™) to distinguish dead and live cells. Data were acquired using a LSR Fortessa™ flow cytometer (BD Biosciences). MFC analysis for samples from the cohort #2 and

#3 (Supplemental Table 1) with combination #2 and #3 of antibodies (Supplemental Table 2) were performed on fresh BM specimens. BM purity was determined by the method of Holdrinet *et al* [1] and diluted samples (purity<50%) were excluded. Staining was performed within 24h following aspiration on 2 million BM cells after one wash of whole BM in Dulbecco's phosphate buffered-saline (PBS, Eurobio). Cells were stained with antibodies for 20mn. Red blood cells were lysed with 1 mL Versalyse™ (Beckman Coulter) and the samples washed once in PBS. Cells were then resuspended in 500µL of PBS and data were acquired using a Navios flow cytometer (Beckman Coulter) with 10 colors, 3 lasers (5+3+2 configuration). Sensitivity of the flow cytometer was daily controlled using Flow-Set Pro beads (Beckman Coulter). Data were analyzed using Kaluza 2.1 version software (Beckman Coulter) using the gating strategy described in Supplemental Figure 1, Supplemental Figure 3 and Supplemental Figure 7. To ensure a robust calculation of entropy in both CD34+CD38+ and CD34+CD38- compartment of cells, samples with less of 500 cells in the fraction of total CD34+ HSPCs and/or less of 100 cells in one of the two fraction of CD34+CD38- or CD34+CD38+ HSPCs were excluded. The median number of CD34+ HSPCs analyzed was of 6045 (min-max: 1003-192573) and of 12216 (min-max: 845-221903) for samples collected in cohort #2 and cohort #3 respectively.

Genomic testing

We reported all clinically relevant variants with a VAF cut-off at 2%. Libraries were prepared using Ampliseq System, according to the manufacturer's instructions, and sequenced on an ion torrent S5XL (Thermo Fisher Scientific, Inc., Waltham, MA, USA). Average coverage per gene was ≥ 600X. Reads were aligned against human genome build 19 (hg19) and analyzed for single nucleotid variant (SNV) calling with NextGENe software (SoftGenetics, Chicago, IL) and with an in-house pipeline (Polydiag, Institut Imagine, Université de Paris). IPSS-M was calculated as previously described with missing data for 2 main effects genes (NPM1 and MLL^{PTD}) and for 4 residuals genes (CEBPA, GNB1, NF1 and PRPF8).[2]

SUPPLEMENTAL FIGURES

Supplemental Figure 1

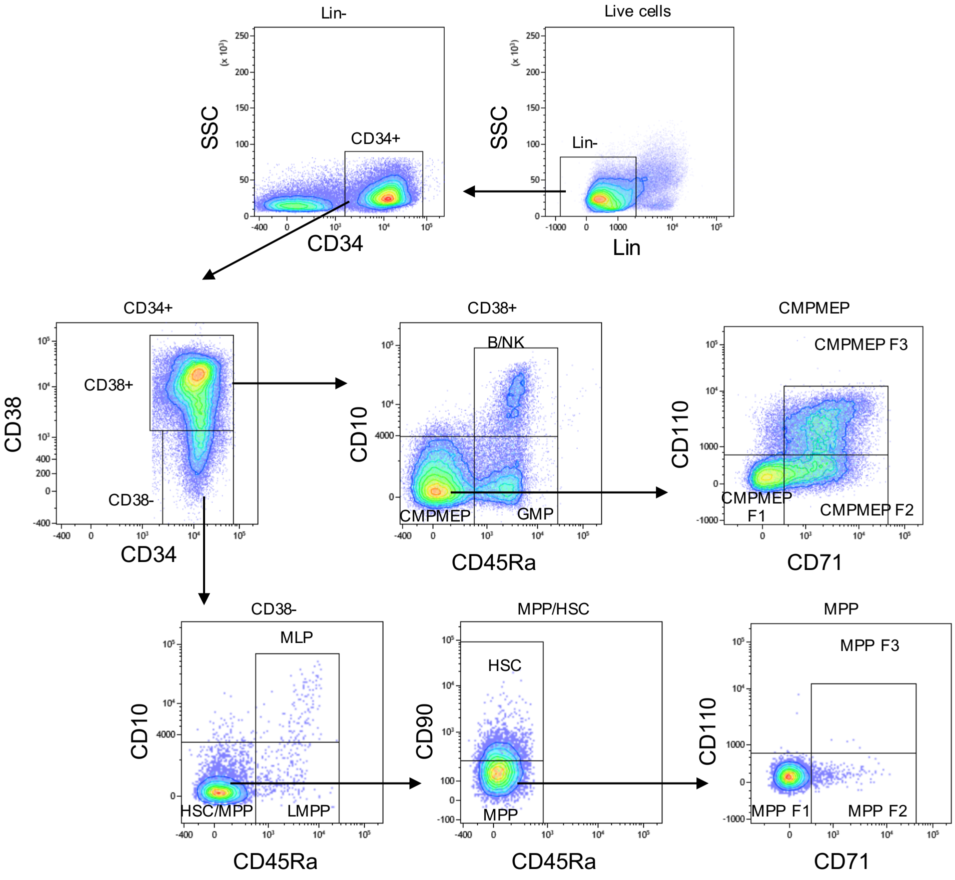

Dussiau C *et al*

Supplemental Figure 1: Different sub-populations of HSPCs identified by MFC with combination #1 (cohort #1). After elimination of lin+ cells (CD235/CD16/CD14/CD11b/CD4/CD8/CD3/CD2) CD34+CD38- and CD34+CD38+ cells were selected according their expression of CD38. Into the CD34+CD38- cells, HSC (CD45Ra- CD10- CD90+), MPP (CD45Ra- CD10- CD90-), LMPP (CD45Ra+ CD10-) and MLP (CD45Ra+ CD10+) populations were identified on a CD45Ra/CD10 plot. The MPP population was then separated into 4 different fractions of cells according their CD71 and CD110 expression: MPP F1 (CD71- CD110-), MPP F2 (CD71+ CD110-), MPP F3 (CD71+ CD110+) and MPP F4 (CD71- CD110+). Into the CD34+CD38+ cells, B/NK progenitors (CD45Ra+ CD10+), GMP (CD45Ra+ CD10-) and CMP/MEP cells (CD45Ra-) were identified on a CD45Ra/CD10 plot. Finally, the CMP/MEP population was separated into 4 different fractions of cells according their CD71 and CD110 expression: CMP/MEP F1 (CD71- CD110-), CMP/MEP F2 (CD71+ CD110-), CMP/MEP F3 (CD71+ CD110+) and CMP/MEP F4 (CD71- CD110+). Figure was obtained from analysis of the sample CTRL1.

Supplemental Figure 2

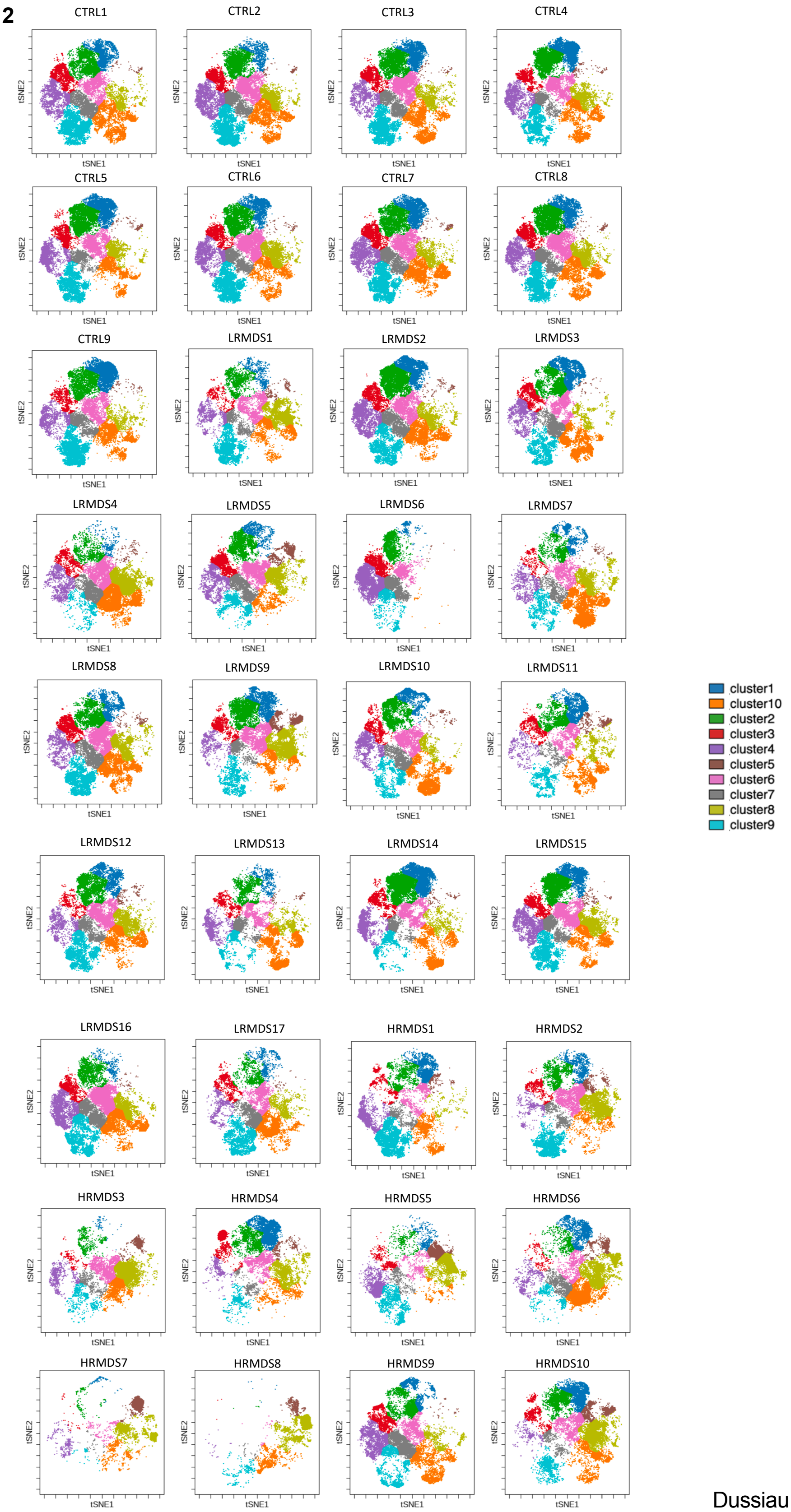

Dussiau C *et al*

Supplemental Figure 2: CD34+ clusters repartition on a t-SNE plot from all control samples and MDS samples analyzed from cohort #1. Among 17 patients with low-risk MDS, 6 (LRMDS4, LRMDS5,

LRMDS6, LRMDS11, LRMDS13, LRMDS14) were identified to have a distinct patterns of HSPCs repartition compared to control. CTRL, control samples; LRMDS, low-risk MDS; HRMDS, high-risk MDS.

Supplemental Figure 3

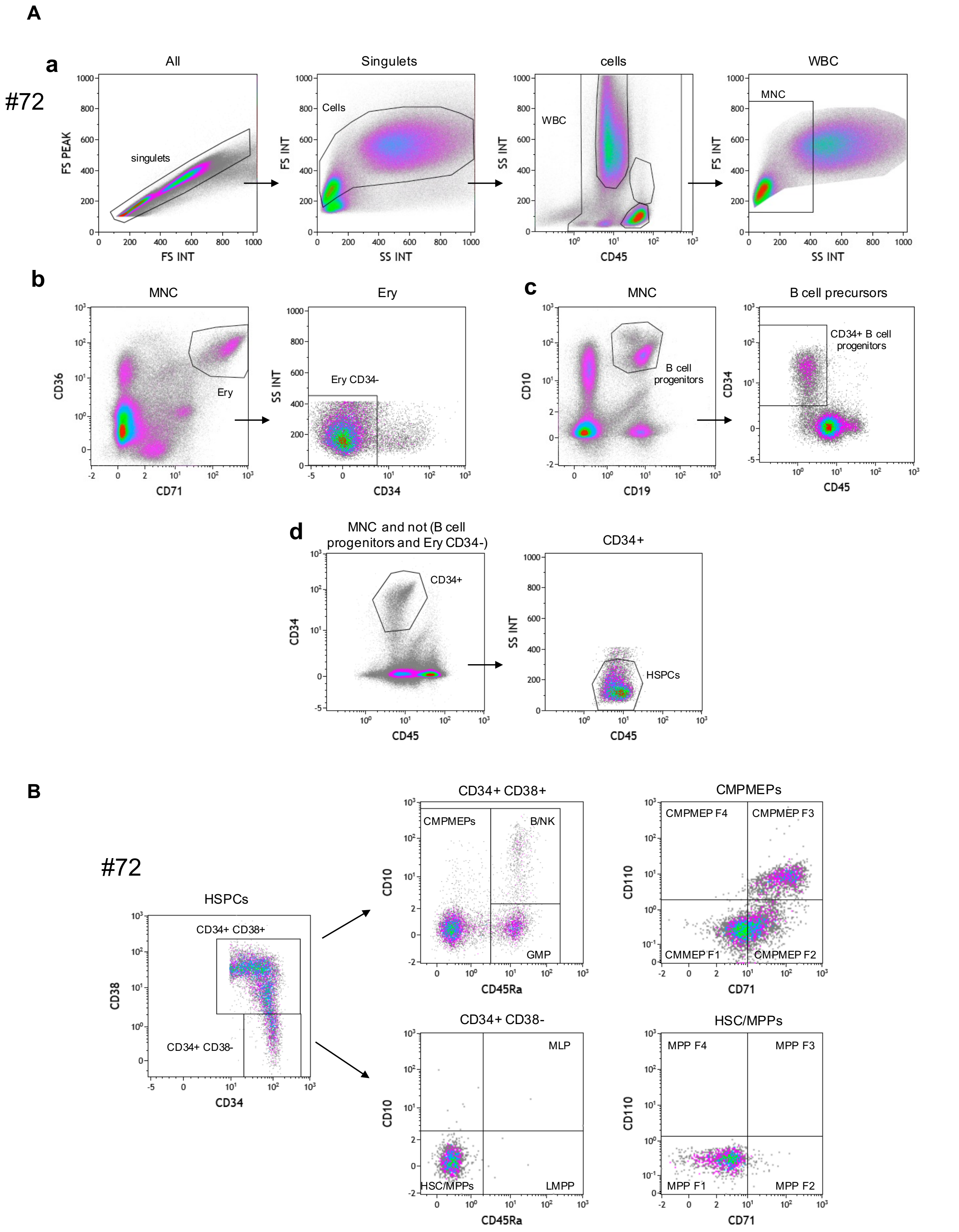

Dussiau C *et al*

Supplemental Figure 3: Gating strategy for identification of CD34+CD38+ and CD34+CD38- HSPCs by MFC with combination #2 (cohort #2). **(A)** Gating of the CD34+ HSPCs. After elimination of doublets, white blood cells (WBC) leucocytes were gated according to the SSC/FSC plot and CD45 intensity in a CD45/SSC plot and Mononuclear cells (MNC) were selected on the SSC/FSC plot (panel a). Residual CD34 negative erythroblastic precursors were selected according their strong expression of CD71 and CD36 on a CD71/CD36 plot and adjusted in the CD34/SSC plot (panel b). B cell precursors were selected on a CD19/CD10 plot and CD34 positive cells were identified on a CD45/CD34 plot (panel c). Then, a boolean gate allows the elimination of the previously identified B cell precursors and CD34- erythroblastic precursors into the MNC population. Into this gate, CD34 positive cells were selected on a CD45/CD34 plot and adjusted in the CD45/SSC plot (panel d). Figures were obtained from analysis of the non-MDS BM sample #72. **(B)** Identification of HSPCs sub-populations. CD34+CD38- and CD34+CD38+ cells were selected according their expression of CD38. Into the CD34+CD38- cells, HSC/MPP (CD45Ra- CD10-), LMPP (CD45Ra+ CD10-) and MLP (CD45Ra+ CD10+) populations were identified on a CD45Ra/CD10 plot. The HSC/MPP population was then separated into 4 different fractions of cells according their CD71 and CD110 expression: MPP F1 (CD71- CD110-), MPP F2 (CD71+ CD110-), MPP F3 (CD71+ CD110+) and MPP F4 (CD71- CD110+). Into the CD34+CD38+ cells, B/NK progenitors (CD45Ra+ CD10+), GMP (CD45Ra+ CD10-) and CMP/MEP cells (CD45Ra-) were identified on a CD45Ra/CD10 plot. Finally, the CMP/MEP population was separated into 4 different fractions of cells according their CD71 and CD110 expression: CMP/MEP F1 (CD71- CD110-), CMP/MEP F2 (CD71+ CD110-), CMP/MEP F3 (CD71+ CD110+) and CMP/MEP F4 (CD71- CD110+). In conclusion, this gating strategy allowed the quantification of the CD34+ population (without B cells precursors) into the leucocytes population and of 13 sub-populations: MPP F1, MPP F2, MPP F3, MPP F4, LMPPs, MLPs, B/NK precursors, GMPs, CMPMEP F1, CMPMEP F2, CMPMEP F3, CMPMEP F4 and CD34+CD38-CD45Ra-CD10+ cells. Quantification of the CD34+ B cells precursors into the global CD34+ cells and of CD34+ HSPCs among WBC was also performed. Figures were obtained from analysis of the non-MDS BM sample #72.

Supplemental Figure 4

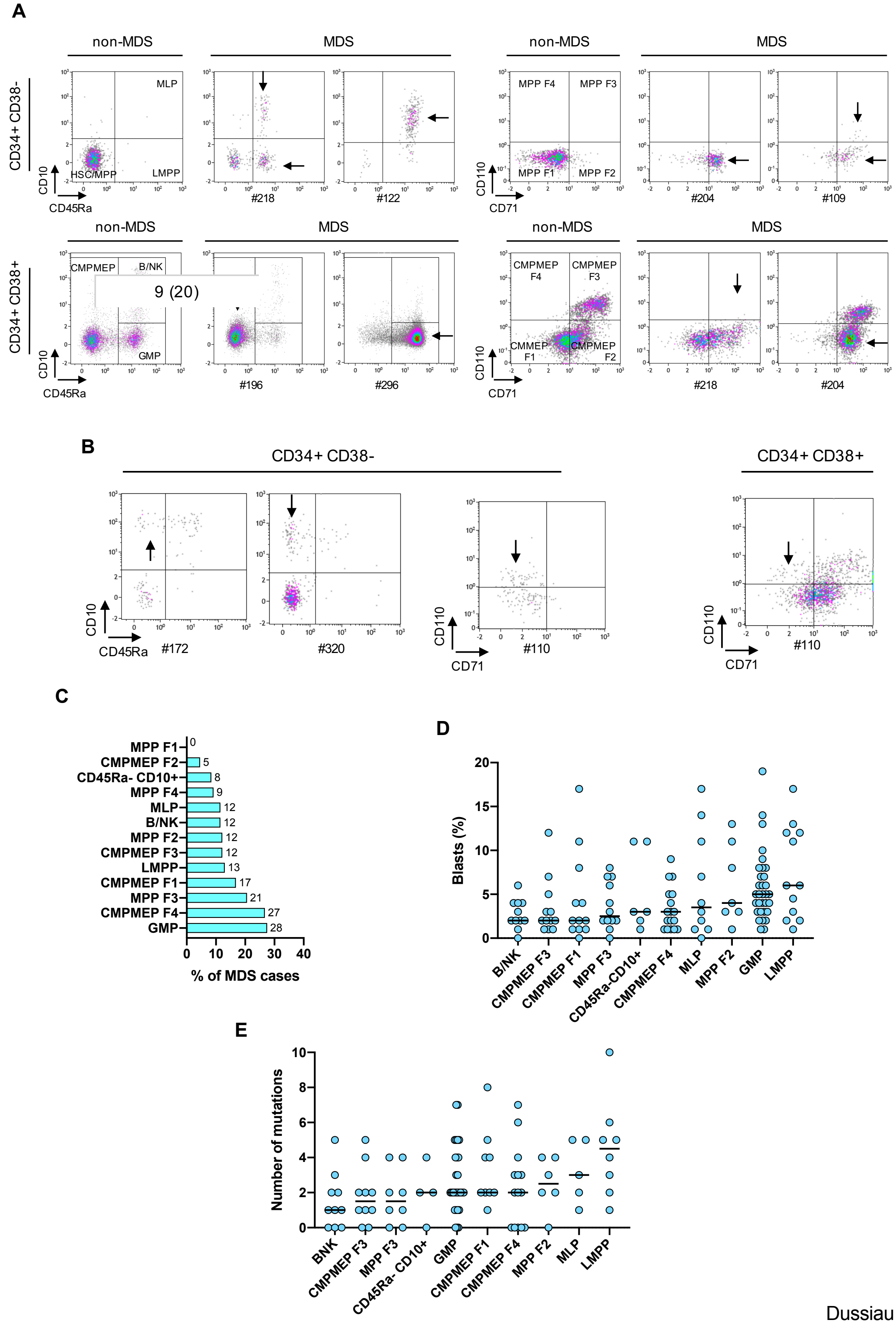

Dussiau C *et al*

Supplemental Figure 4: Examples of abnormal patterns of HSPCs repartition detected in MDS samples compared to non-MDS samples and correlation with MDS characteristics. BM samples

are from cohort #2. **(A)** Upper left panel: increase of MLP (patient #122, IPSS-R high) and of LMPP (patient #218, IPSS-R low). Upper right panel: increase of MPP F2 (patient #204, IPSS-R high) and of MPP F3 (patient #109, IPSS-R very high). Down left panel: increase of CMPMEP (patient #196, IPSS-R low) or GMP (patient #296, IPSS-R intermediate). Down right panel: decrease of CMPMEP F3 (patient #218, IPSS-R low) or increase of CMPMEP F2 (patient #204, IPSS-R high). **(B)** Examples of abnormal HSPCs sub-populations detected in MDS samples but not in of non-MDS samples without cytopenia such as CD34+ CD38- CD45Ra- CD10+ cells (patients #172, IPSS-R low and #320, IPSS-R intermediate); MPP F4 and CMPMEP F4 (patient #110, IPSS-R very high). **(C)** Proportion of MDS cases with increased of each HSPCs sub-populations determined according the calculation of z-score >2 compared to the cohort of non-MDS samples without cytopenia. **(D)** BM Blast cells percentage according the pattern of HSPCs repartition. Lines represent medians. **(E)** Number of mutations according the pattern of HSPCs repartition. Lines represent medians.

Supplemental Figure 5

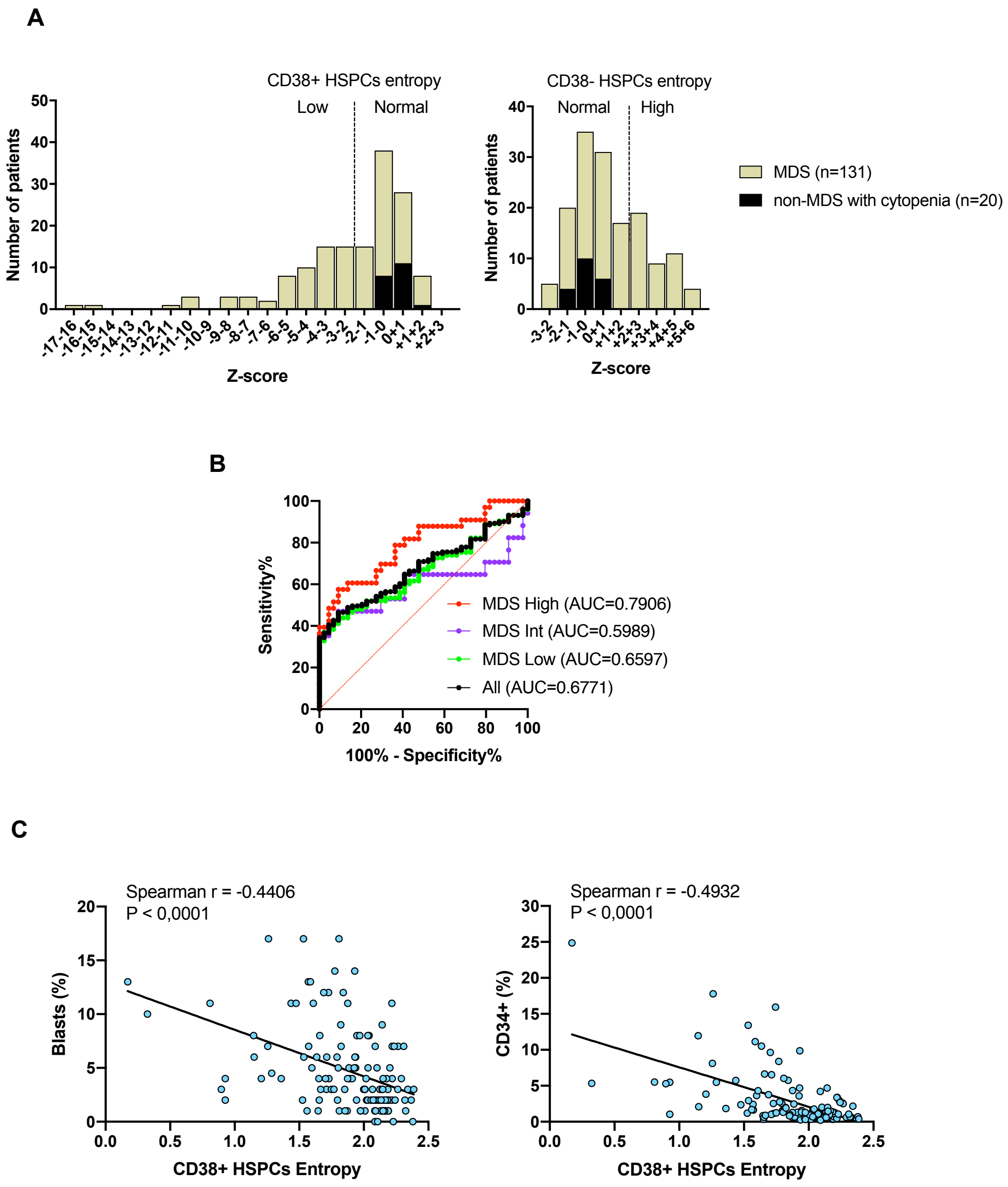

Dussiau C *et al*

Supplemental Figure 5: Detection of MDS patients with abnormal level of CD38+ or CD38-HSPCs entropy. BM samples are from cohort #2. **(A)** Repartition of patients (MDS, n=131 and non-MDS with

cytopenia, n=20) according their calculated z-score compared to the cohort of non-MDS samples without cytopenia for CD38+ (left panel) and CD38- (right panel) HSPCs entropy. **(B)** ROC curve for the CD38-HSPCs entropy in the whole cohort of patients with MDS (black curve, n=131), or MDS low risk (very low/low IPSS-R; orange curve; n=73) or MDS intermediate risk (blue curve; n=17) or MDS high risk (high/very high IPSS-R; red curve; n=33) compared to non-MDS samples with or without cytopenia (n=44). **(C)** Correlation between level of CD38+HSPCs entropy and blast percentage detected by morphology (left panel) or quantification of CD34+ cells by MFC (right panel). Correlation was determined using non-parametric Spearman correlation test.

Supplemental Figure 6

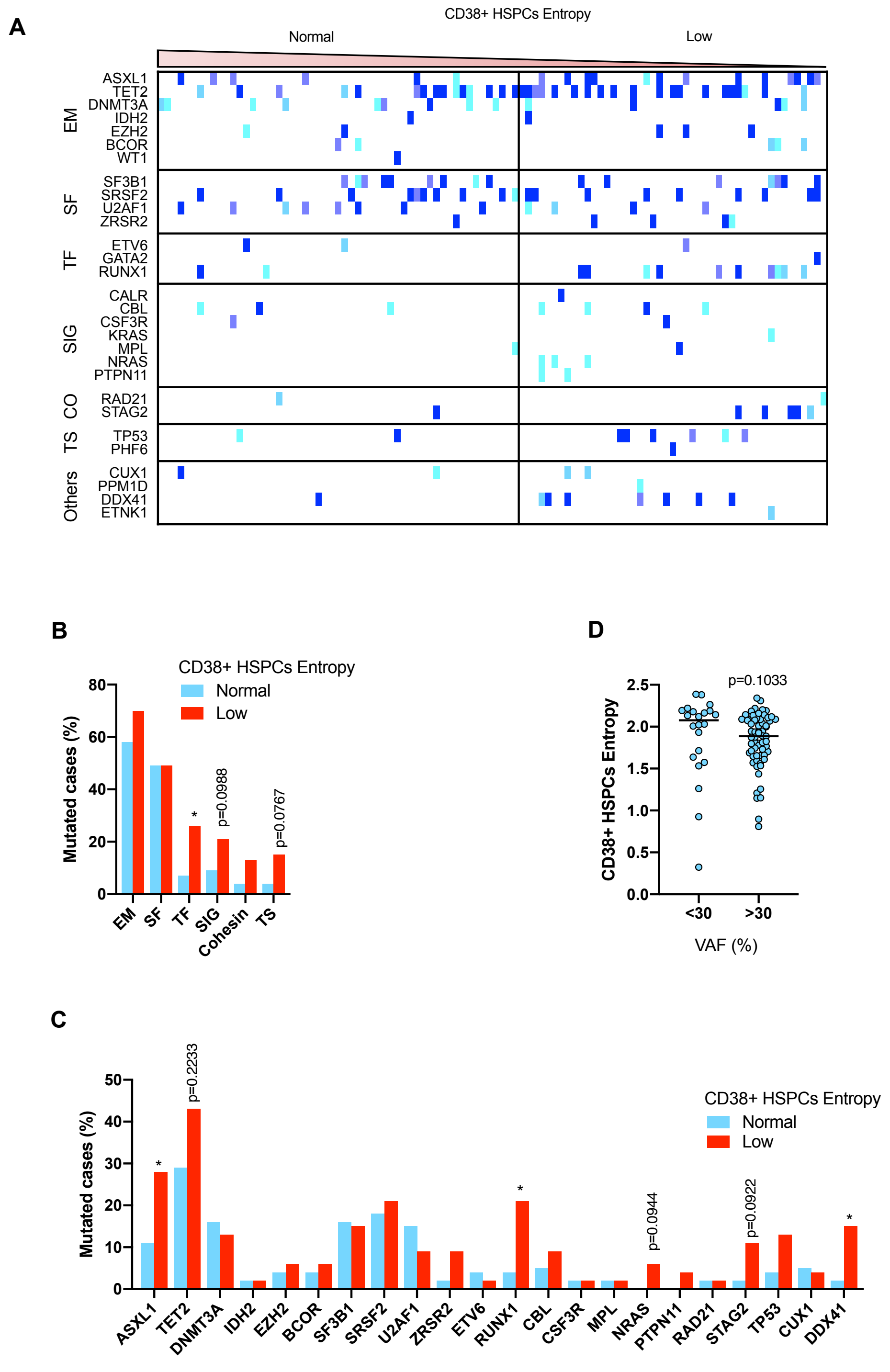

Dussiau C *et al*

Supplemental Figure 6: Genomic landscape of MDS patients stratified according their level of CD38+ HSPCs entropy. BM samples are from cohort #2. **(A)** Mutational landscape of the 102 MDS

samples according the level of the CD38+HSPCs entropy (normal or low). Abnormal level of CD38+HSPCs entropy was determined according the calculation of z-score ≤-2 compared to the cohort of non-MDS samples without cytopenia. Patients are shown according their CD38+HSPCs entropy value. Mutational VAF is coded; ice, 2-10%; sky, 10-20%; orchid, 20-30%; blueberry, >30%. If multiple mutations are present in a case, the highest VAF is shown. **(B)** Prevalence of somatic mutations in mains groups of genes in BM from MDS patients with normal (n=55) or low (n=47) level of CD38+HSPCs entropy. Low level of CD38+HSPCs entropy was determined according the calculation of z-score ≤-2 compared to the cohort of non-MDS samples without cytopenia. Statistical significance was calculated using Fisher's exact tests. EM, Epigenetic Modifiers; SF, Splicing Factors; SIG, Signaling; TF, Transcription Factors; TS, Tumor Suppressors. **(C)** Prevalence of somatic mutations in oncogenes and leukemia-relevant genes in BM from MDS patients with normal (n=55) or low (n=47) CD38+HSPCs entropy. Genes mutated in ≥2 patients are shown. Low level of CD38+HSPCs entropy was determined according the calculation of z-score ≤-2 compared to the cohort of non-MDS samples without cytopenia. Statistical significance was calculated using Fisher's exact tests. **(D)** Levels of CD38+HSPCs entropy according the VAF (< or ≥30%) of somatic mutation detected in mutated MDS samples (n=85/102). In cases with multiple mutations, the highest VAF was used. Lines represent means ± s.d. Statistical significance was determined using unpaired two-tailed Mann-Whitney tests.

Supplemental Figure 7

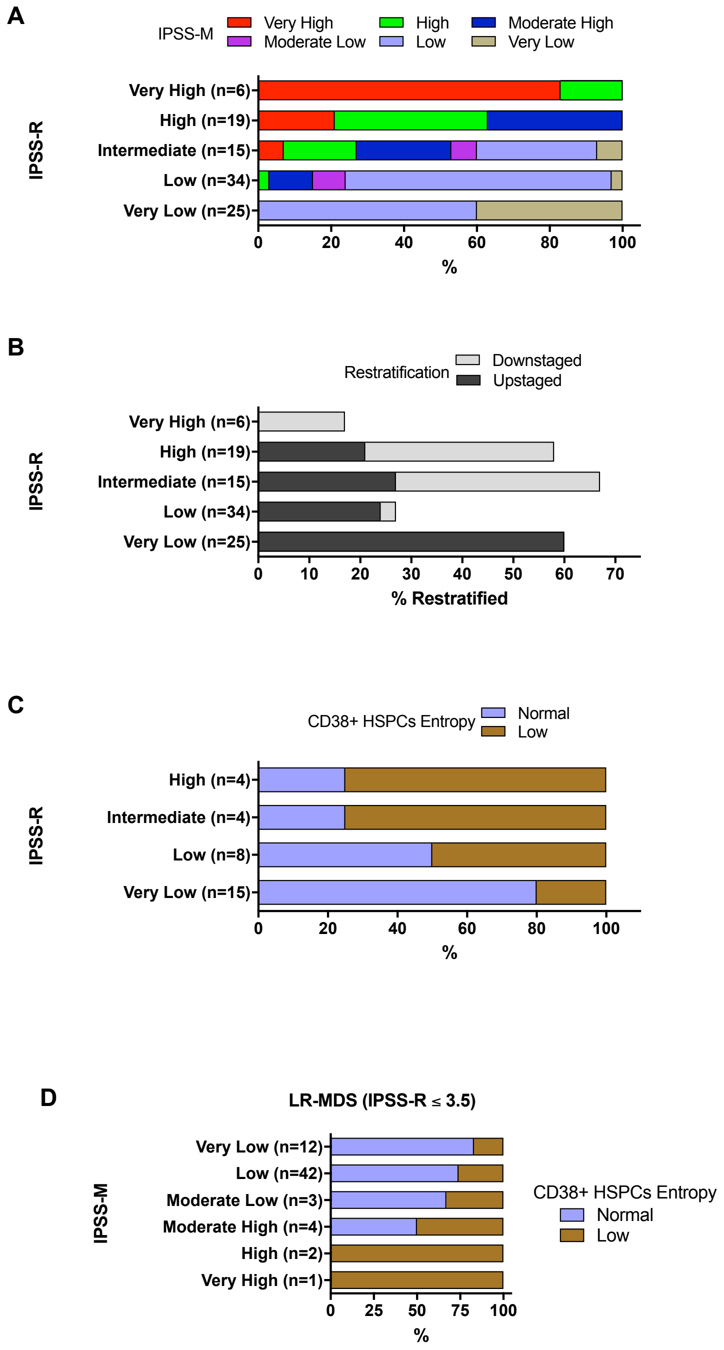

Dussiau C *et al*

Supplemental Figure 7: Correlation of IPSS-M with the level of CD38+ HSPCs entropy. BM samples are from cohort #2. **(A)** Restratification of IPSS-R to IPSS-M (n=99). Each row corresponds to

one IPSS-R category, and colors represent the IPSS-M categories. **(B)** Proportion of restratified patients in each IPSS-R stratum (counting any shift). **(C)** Proportion of IPSS-M-restratified upstaged patients with either normal or low level of CD38+ HSPCs entropy for each group of IPSS-R. **(D)** Association between the level of CD38+ HSPCs entropy and restratification of patients with LR-MDS based on their IPSS-R ≤3.5 (n=64).

Supplemental Figure 8

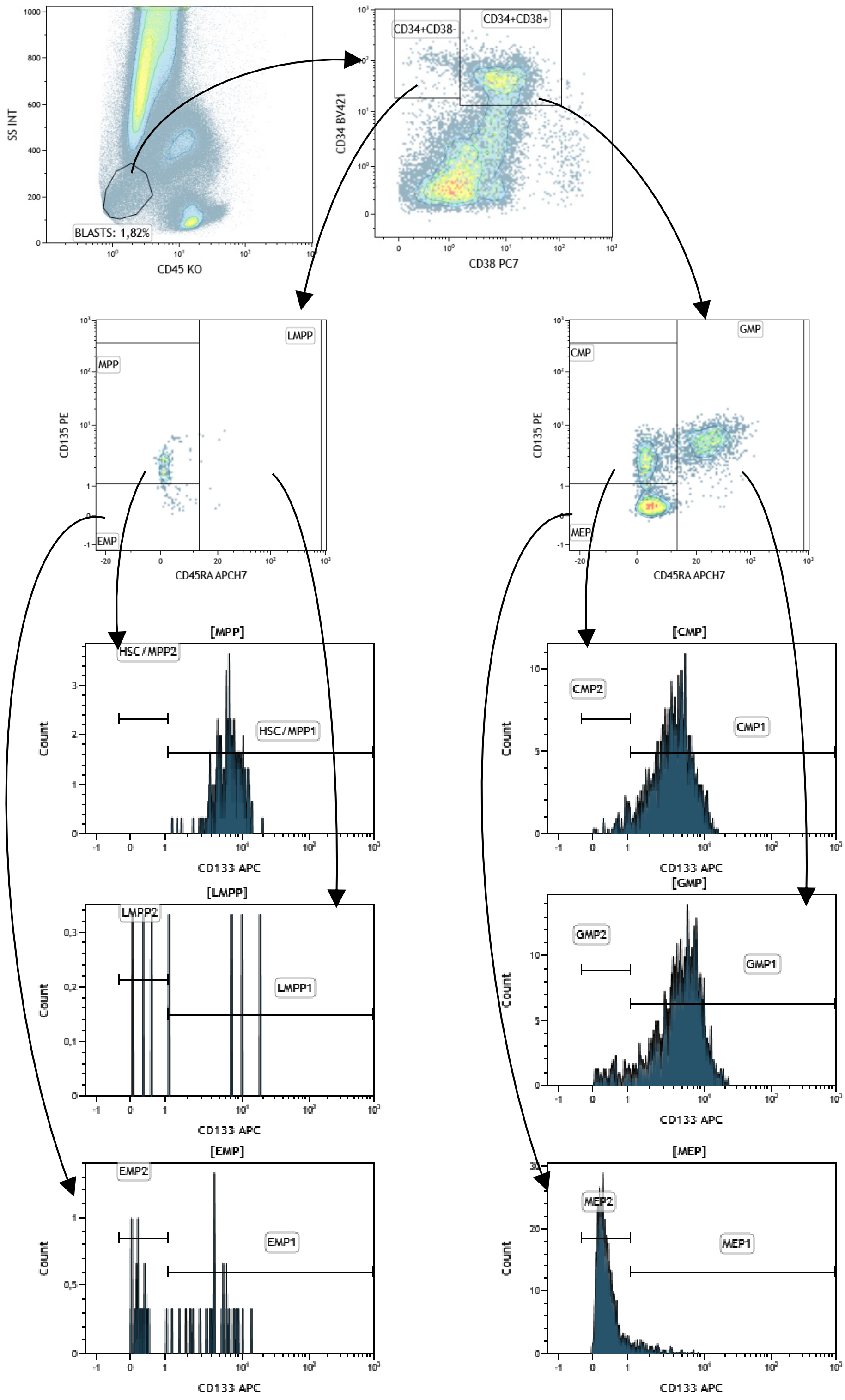

Dussiau C *et al*

Supplemental Figure 8: gating strategy for identification of CD34+CD38+ and CD34+CD38- HSPCs by MFC with combination #3 (cohort #3). Gating of the CD34+ HSPCs: after elimination of

doublets, white blood cells (WBC) leucocytes were gated according to the SSC/FSC plot and CD45 intensity in a CD45/SSC plot and Mononuclear cells (MNC) were selected on the SSC/FSC plot. Blasts cells were selected on a CD45/SSC plot. Then, CD34+ CD38- and CD34+ CD38+ populations were selected according to their expression of CD38, adjusted to a threshold defined by the lymphocytes, on a CD34/CD38 plot. Identification of HSPCs sub-populations: into the CD34+CD38- stem cell population, HSC/MPP (CD45Ra- CD135+), LMPP (CD45Ra+ CD135+) and EMP (CD45Ra- CD135-) populations were identified on a CD45Ra/CD135 plot. These populations were then separated into 2 different fractions of cells according their CD133 expression: MPP F1 (CD133+), MPP F2 (CD133-), LMPP F1 (CD133+), LMPP F2 (CD133-), EMP F1 (CD133+) and EMP F2 (CD133-). Into the CD34+CD38+ progenitor cell population, CMP (CD45Ra+ CD135-), GMP (CD45Ra+ CD135+) and MEP cells (CD45Ra- CD135-) were identified on a CD45Ra/CD135 plot. Finally, these population were separated into 2 different fractions of cells according their CD133 expression: CMP F1 (CD133+), CMP F2 (CD133-), GMP F1 (CD133+), GMP F2 (CD133-), MEP F1 (CD133+) and MEP F2 (CD133-). In conclusion, this gating strategy allowed the quantification of the CD34+ population into the leucocytes population and of 12 sub-populations. Figures were obtained from analysis of the MDS BM sample #140.

Supplemental Figure 9

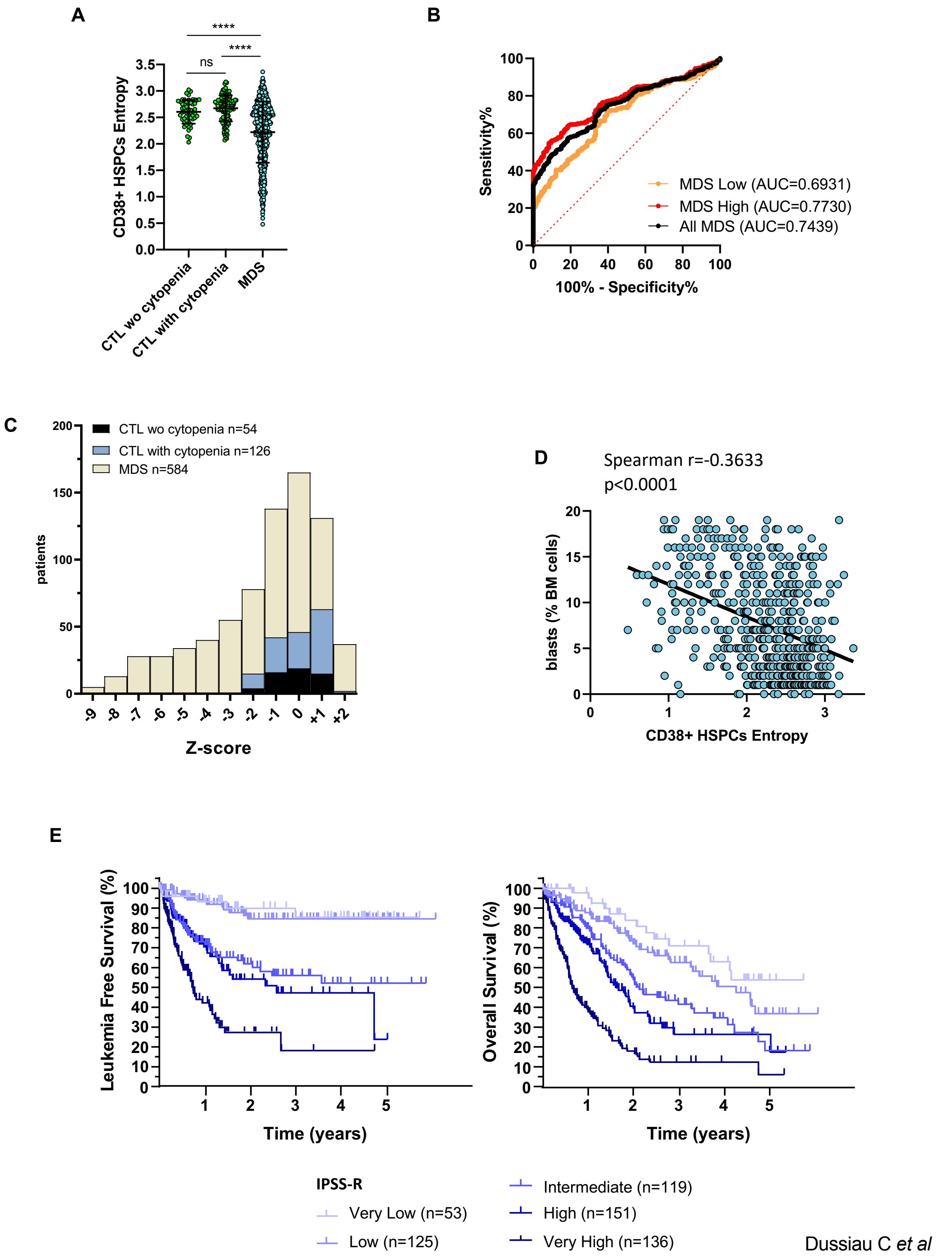

Dussiau C *et al*

Supplemental Figure 9: Analysis of CD38+HSPCs entropy in samples from cohort #3. (A) CD38+HSPCs levels in non-MDS samples with (n=126) or without (n=54) cytopenia and in MDS

samples (n=584). Lines represent means ± s.d. Statistical significance was determined using unpaired two-tailed Mann-Whitney tests. **(B)** ROC curve for the CD38+HSPCs entropy in the whole cohort of patients with MDS (black curve, n=584), or MDS low risk (IPSS-R≤3.5; orange curve; n=318) or MDS high risk (IPSS-R>3.5; red curve; n= 266) compared to non-MDS samples with or without cytopenia (n=180). **(C)** Repartition of non-MDS samples without cytopenia and MDS samples according their level of CD38+HSPCs entropy. **(D)** Correlation between level of CD38+HSPCs entropy and blast percentage detected by morphology. Correlation was determined using non-parametric Spearman correlation test. **(E)** Overall survival and leukemia free survival of MDS patients stratified by their IPSS-R risk.

Supplemental Figure 10

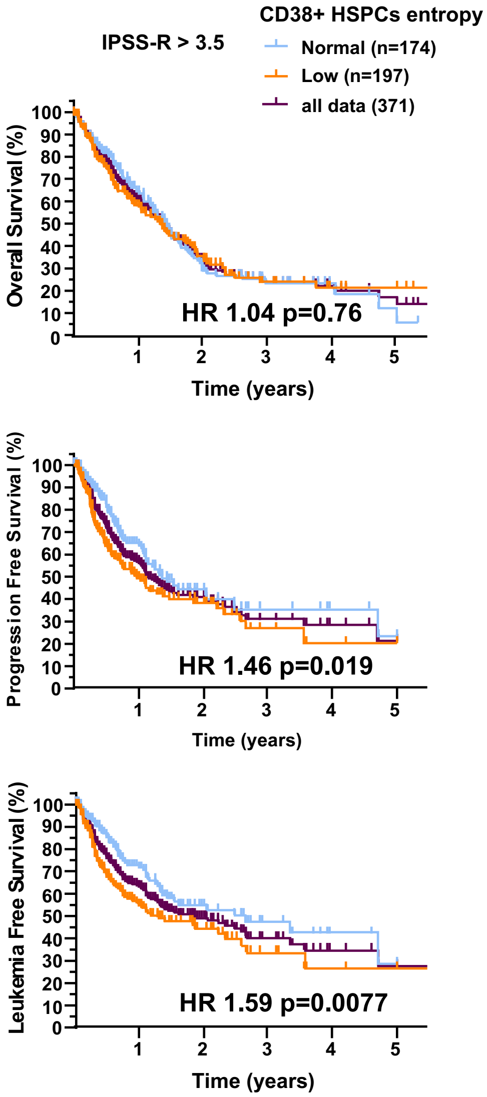

Dussiau C *et al*

Supplemental Figure 10: Kaplan-Meier estimates of survival outcomes of patients with HR-MDS

according to CD38+ HSPCs entropy level. BM samples are from cohort #3. Patients with HR-MDS were defined based on their IPSS-R>3.5 (n=371).

SUPPLEMENTAL TABLES

		Institution #1		Institution #2
		cohort #1	cohort #2	cohort #3
Samples	**Patients**	n= 36	n=184	n=764
	MDS	n=27	n=131	n=584
	AML		n=9	
	**Controls**	n=9	n=44	n=180
	without cytopenia	n=9	n=20	n=54
	with cytopenia	n=0	n=24	n=126
MFC	**Antibodies**	Combination #1 (19 markers)	Combination #2 (9 markers)	Comibination #3 (7 markers)
	**Sample type**	Thawed MNC CD34+ sorted cells from BM (MDS) or from bone of the femoral head (CTL)	Fresh BM	Fresh BM
	**Gating strategy**	Unsupervised	Manual	Manual

Supplemental Table 1: repartition of samples collected in the study

AML with t(8;21), n=2; AML myelodysplasia related, n=3; AML post cytotoxic therapy, n=1 and AML defined by differentiation, n=3.

Combination #1			Combination #2			Combination #3		
**Marker & Fluorochrom**	**Clone**	**Supplier**	**Marker & Fluorochrom**	**Clone**	**Supplier**	**Marker & Fluorochrom**	**Clone**	**Supplier**
**CD34 PC7**	581	Beckman Coulter	**CD34 AA700**	581	Beckman Coulter	**CD34 BV421**	581	Becton Dickinson
**CD38 PECF564**	HIT2	Becton Dickinson	**CD38 PC7**	HB-7	Becton Dickinson	**CD38 PC7**	HB-7	Becton Dickinson
**CD90 BV421**	5E10	Becton Dickinson	**CD45 KrOr**	J33	Beckman Coulter	**CD45 KrOr**	J33	Beckman Coulter
**CD45Ra BUUV737**	HI100	Becton Dickinson	**CD45Ra BV421**	HI100	Becton Dickinson	**CD45Ra APCH7**	HI100	Becton Dickinson
**CD71 BV786**	M-A712	Becton Dickinson	**CD71 APC-Vio770**	AC102	Miltenyi Biotec	**CD71 FITC**	M-A712	Becton Dickinson
**CD110 PE**	BAH-1	Becton Dickinson	**CD110 PE**	BAH-1	BD Pharmingen	**CD135 PE**	4G8	Becton Dickinson
**CD10 PerCP CY5.5**	REA877	Miltenyi Biotec	**CD10 APC**	REA877	Miltenyi Biotec	**CD133 APC**	AC133	Miltenyi Biotec
**CD123 BV711**	9F5	Becton Dickinson	**CD19 ECD**	J3-119	Beckman Coulter			
**CD19 FITC**	4G7	Becton Dickinson	**CD36 FITC**	FA6,152	Beckman Coulter			
**CD49f AF647**	GoH3	Becton Dickinson						
**CD7 APC Vio770**	6B7	Miltenyi Biotec						
**CD2 FITC**	S5.2	Becton Dickinson						
**CD3 FITC**	SK7	Becton Dickinson						
**CD4 FITC**	SK3	Becton Dickinson						
**CD8 FITC**	SK1	Becton Dickinson						
**CD11b FITC**	ICRF44	Becton Dickinson						
**CD14 FITC**	MφP9	Becton Dickinson						
**CD16 FITC**	NKP15	Becton Dickinson						
**CD235a FITC**	GA-R2	Becton Dickinson						

Supplemental Table 2: List of antibodies used for identification of HSPCs sub-populations by MFC.

**Combination # 1**	**CD34+ Lin-**	**CD38-**	Hematopietic Stem Cells (**HSCs**)	CD45Ra- CD10- CD90+		
			Multilymphoid Progenitors (**MLP**)	CD45Ra+ CD10+ CD90-		
			Lymphoid-Primed Multipotent Progenitors (**LMPP**)	CD45Ra+ CD10- CD90-		
			Multipotent Progenitors Populations (**MPPs**)	CD45Ra- CD10- CD90-	**MPP F1**	CD71- CD110-
					**MPP F2**	CD71+ CD110-
					**MPP F3**	CD71+ CD110+
		**CD38+**	**B/NK Progenitors**	CD45Ra+ CD10+		
			Granulocyte-Monocyte Progenitor (**GMPs**)	CD45Ra+ CD10-		
			Common Myeloid Progenitor (**CMP**) / Megakaryocyte-Erythroid Progenitors (**MEP**)	CD45Ra- CD10-	**CMPMEP F1**	CD71- CD110-
					**CMPMEP F2**	CD71+ CD110-
					**CMPMEP F3**	CD71+ CD110+

**Combintion #2**	**CD45low SSCint CD34+**	**CD38-**	Multilymphoid Progenitors (**MLP**)	CD45Ra+ CD10+		
			Lymphoid-Primed Multipotent Progenitors (**LMPP**)	CD45Ra+ CD10-		
			Hematopietic Stem Cells (HSCs)/ Multipotent Progenitors Populations (**MPPs**)	CD45Ra- CD10-	**MPP F1**	CD71- CD110-
					**MPP F2**	CD71+ CD110-
					**MPP F3**	CD71+ CD110+
		**CD38+**	**B/NK Progenitors**	CD45Ra+ CD10+		
			Granulocyte-Monocyte Progenitor (**GMPs**)	CD45Ra+ CD10-		
			Common Myeloid Progenitor (**CMP**) / Megakaryocyte-Erythroid Progenitors (**MEP**)	CD45Ra- CD10-	**CMPMEP F1**	CD71- CD110-
					**CMPMEP F2**	CD71+ CD110-
					**CMPMEP F3**	CD71+ CD110+

**Combination #3**	**CD45low SSCint CD34+**	**CD38-**	Hematopoietic Stem Cells (**HSCs**)/Multipotent Progenitors (**MPP**)	CD45Ra-CD135+	**HSC/MPP F1**	CD133+
					**HSC/MPP F2**	CD133-
			Lymphoid-Primed Multipotent Progenitors (**LMPP**)	CD45Ra+CD135+	**LMPP F1**	CD133+
					**LMPP F2**	CD133-
			Erythro-Myeloid Progenitors (**EMP**)	CD45Ra-CD135-	**EMP F1**	CD133+
					**EMP F2**	CD133-
		**CD38+**	Common Myeloid Progenitors (**CMP**)	CD45Ra-CD135+	**CMP F1**	CD133+
					**CMP F2**	CD133-
			Granulocyte-Monocyte Progenitors (**GMP**)	CD45Ra+CD135+	**GMP F1**	CD133+
					**GMP F2**	CD133-
			Megacaryocyte-Erythroid Progenitors (**MEP**)	CD45Ra-CD135-	**MEP F1**	CD133+
					**MEP F2**	CD133-

Supplemental Table 3: Detailed phenotype of the different CD34+ HSPCs sub-populations identified by different MFC strategies.

The phenotype of all HSPCs subpopulations were previously described. [3–5]

	CTL without cytopenia (n=9)	MDS (n=27)
**Age, y , median (range)**	68 (45-83)	77 (55-94)
**Males, n (%)**	2 (22)	13 (48)
**Hb, g/dL, median (range), [NA]**		10,4 (7,6-14) [2]
**ANC, x 10^9/L, median (range), [NA]**		2,515 (0,39-35,93) [3]
**Plt, x 10^9/L, median (range), [NA]**		139 (10-717) [3]
**WHO 2022, n (%)**		
MDS-LB		11 (41)
MDS-LB-RS		2 (7)
MDS-5q		1 (4)
MDS-IB1		7 (26)
MDS-IB2		6 (22)
**IPSS-R, n (%)**		**n= 23 (85) ***
Very Low		4 (17)
Low		6 (26)
Intermediate		6 (26)
High		4 (17)
Very High		3 (13)

Supplemental Table 4: Characteristics of patients analyzed by MFC with combination #1 (Cohort #1).

*IPSS-R was unavailable due to karyotype failure (n=4).

ANC, absolute neutrophil count; Hb, hemoglobin, MDS-5q, MDS with low blasts and with isolated del(5q); MDS-IB1/2, MDS with increased blasts 1/2; MDS-LB, MDS with low blasts; MDS-LB-RS, MDS with low blasts and ring sideroblasts; NA, not available; Plt, Platelet.

		Mean (%)	Lower 95% CI of mean	Upper 95% CI of mean
CD34+ CD38-	MPP F1	84,60	76,93	92,27
	MPP F2	5,17	3,15	7,20
	MPP F3	0,06	-0,01	0,13
	MPP F4	0,51	0,12	0,89
	CD45Ra- CD10+	1,34	0,67	2,01
	LMPP	4,96	-0,59	10,51
	MLP	3,36	0,98	5,74
CD34+ CD38+	B/NK	7,49	6,58	8,41
	GMP	29,55	26,09	33,00
	CMPMEP F1	18,79	15,62	21,95
	CMPMEP F2	25,62	22,58	28,66
	CMPMEP F3	17,82	15,50	20,14
	CMPMEP F4	0,73	0,47	0,99

Supplemental Table 5: **Quantification of the different sub-populations in non-MDS samples without cytopenia collected in cohort #2, among CD34+CD38- or CD34+CD38+HSPCs.** These values were used as references for normal frequency of each HSPCs sub-populations.

REFERENCES

1. Holdrinet RS, von Egmond J, Wessels JM, Haanen C. A method for quantification of peripheral blood admixture in bone marrow aspirates. *Exp. Hematol.* 1980;8(1):103–107.

2. Bernard Elsa, Tuechler Heinz, Greenberg Peter L., et al. Molecular International Prognostic Scoring System for Myelodysplastic Syndromes. *NEJM Evid.* 2022;1(7):EVIDoa2200008.

3. Notta F, Zandi S, Takayama N, et al. Distinct routes of lineage development reshape the human blood hierarchy across ontogeny. *Science*. 2016;351(6269):aab2116.

4. Karamitros D, Stoilova B, Aboukhalil Z, et al. Single-cell analysis reveals the continuum of human lympho-myeloid progenitor cells. *Nat. Immunol.* 2018;19(1):85–97.

5. Görgens A, Radtke S, Möllmann M, et al. Revision of the human hematopoietic tree: granulocyte subtypes derive from distinct hematopoietic lineages. *Cell Rep.* 2013;3(5):1539–1552.
